# Comprehensive Insights Into Composition, Metabolic Potentials, and Interactions Among Archaeal, Bacterial, and Viral Assemblages in Meromictic Lake Shunet in Siberia

**DOI:** 10.3389/fmicb.2018.01763

**Published:** 2018-08-20

**Authors:** Yu-Ting Wu, Cheng-Yu Yang, Pei-Wen Chiang, Ching-Hung Tseng, Hsiu-Hui Chiu, Isaam Saeed, Bayanmunkh Baatar, Denis Rogozin, Saman Halgamuge, Andrei Degermendzhi, Sen-Lin Tang

**Affiliations:** ^1^Department of Forestry, National Pingtung University of Science and Technology, Neipu, Taiwan; ^2^Biodiversity Research Center, Academia Sinica, Taipei, Taiwan; ^3^Germark Biotechnology Co., Ltd., Taichung, Taiwan; ^4^Optimisation and Pattern Recognition Group, Melbourne School of Engineering, The University of Melbourne, Melbourne, VIC, Australia; ^5^Molecular and Biological Agricultural Sciences, Taiwan International Graduate Program, Academia Sinica, Taipei, Taiwan; ^6^Department of Life Sciences, Graduate Institute of Biotechnology, National Chung-Hsing University, Taichung, Taiwan; ^7^Institute of Biophysics, Siberian Division of Russian Academy of Sciences, Krasnoyarsk, Russia; ^8^Siberian Federal University, Krasnoyarsk, Russia

**Keywords:** Lake Shunet, metagenomics, archaeal, bacterial and viral assemblages, meromictic lake

## Abstract

Microorganisms are critical to maintaining stratified biogeochemical characteristics in meromictic lakes; however, their community composition and potential roles in nutrient cycling are not thoroughly described. Both metagenomics and metaviromics were used to determine the composition and capacity of archaea, bacteria, and viruses along the water column in the landlocked meromictic Lake Shunet in Siberia. Deep sequencing of 265 Gb and high-quality assembly revealed a near-complete genome corresponding to *Nonlabens* sp. *sh3vir*. in a viral sample and 38 bacterial bins (0.2–5.3 Mb each). The mixolimnion (3.0 m) had the most diverse archaeal, bacterial, and viral communities, followed by the monimolimnion (5.5 m) and chemocline (5.0 m). The bacterial and archaeal communities were dominated by *Thiocapsa* and *Methanococcoides*, respectively, whereas the viral community was dominated by *Siphoviridae*. The archaeal and bacterial assemblages and the associated energy metabolism were significantly related to the various depths, in accordance with the stratification of physicochemical parameters. Reconstructed elemental nutrient cycles of the three layers were interconnected, including co-occurrence of denitrification and nitrogen fixation in each layer and involved unique processes due to specific biogeochemical properties at the respective depths. According to the gene annotation, several pre-dominant yet unknown and uncultured bacteria also play potentially important roles in nutrient cycling. Reciprocal BLAST analysis revealed that the viruses were specific to the host archaea and bacteria in the mixolimnion. This study provides insights into the bacterial, archaeal, and viral assemblages and the corresponding capacity potentials in Lake Shunet, one of the three meromictic lakes in central Asia. Lake Shunet was determined to harbor specific and diverse viral, bacterial, and archaeal communities that intimately interacted, revealing patterns shaped by indigenous physicochemical parameters.

## Introduction

Meromictic lakes have a mixed upper oxic mixolimnion, an interface chemocline, and a lower stagnant anoxic monimolimnion, which does not mix with either of the upper layers (Bowman et al., 2000). Their specific zones having stratified and unique biogeochemical characteristics provide novel opportunities to compare microbial loops and biogeochemical processes among the distinct zones (Bowman et al., 2000; Lauro et al., 2011; Comeau et al., 2012).

Lake Shunet (54° 25′N, 90° 13′E), located in the central Altai–Sayan mountain region in southern Siberia, is one of the only three meromictic lakes in the entire Asian region of Russia. This lake has sharp gradients of salinity, dissolved oxygen, hydrogen sulfide (H_2_S), and a dense population of anoxygenic phototrophic (purple sulfur) bacteria in the chemocline zone, which is comparable to that of Lake Mahoney (Canada), known for having the most purple sulfur bacteria of any lake in the world (Overmann et al., 1991; Rogozin et al., 2012). With its stable stratification, Lake Shunet provides a valuable opportunity for comparative studies on biogeochemistry (Kallistova et al., 2006), physicochemical parameters (Degermendzhy et al., 2010), and microbial communities (Lunina et al., 2007; Rogozin et al., 2009, 2010, 2012). Microscopic techniques, pigment analysis, culture, FISH-TSA techniques and PCR-DGGE have been used to disentangle the seasonal changes of anoxygenic photosynthetic bacterial community, sulfate reduction and methanogenesis and ecophysiology of phototrophic sulfur bacteria at the chemocline of Lake Shunet (Kallistova et al., 2006; Lunina et al., 2007; Rogozin et al., 2009, 2010, 2012). However, these approaches are limited in providing a fine-scale, comprehensive insight into the microbial assemblages and their potential functions. Therefore complete microbial community composition still remain unknown along the three zones. Until now, studies on the makeup of microbial populations and their functional properties in meromictic salt lakes by using whole-genome shotgun approaches have only been undertaken in Antarctica; that is, Ace Lake (Ng et al., 2010; Lauro et al., 2011) and Organic Lake (Yau et al., 2011). Similar to Ace Lake (68° S) (Rankin et al., 1999), Lake Shunet (54°N) is also a saline, high-latitude meromictic lake. Ace Lake is one of the most investigated lakes in Antarctica, in terms of its physicochemical profile, community structure, and functional dynamics (Laybourn-Parry and Bell, 2014). Both lakes are analogous, exhibiting characteristics such as permanent water stratification into three main zones, high H_2_S in the monimolimnion, and a gradient of dissolved oxygen (Table 1) (Lauro et al., 2011; Laybourn-Parry and Bell, 2014).

**Table 1:** Physicochemical parameters of three sampling depths in Lake Shunet.

**Parameters**	**3.0 m**	**5.0 m**	**5.5 m**
Salinity (g L^−1^)	26	40	71
pH	8.1	7.6	6.7
Temp (°C)	15.5	9.5	7.5
O_2_ (mg l^−1^)	10.9	0	0
N_tot_ (mg l^−1^)	2.9	4.6	5
C_tot_ (mg l^−1^)	100.7	160	245
Na^+^ (g l^−1^)	4.8	8.3	11.2
P (mg l^−1^)	0.2	0.5	1.1
H_2_S (mg l^−1^)	0	0	400
${\text{NH}}_{4}^{+}$ (μg l^−1^)	516.7	490	410
${\text{NO}}_{2}^{-}$ (μg l^−1^)	19.2	34	49
${\text{NO}}_{3}^{-}$ (μg l^−1^)	63.8	24	21
${\text{SO}}_{4}^{2-}$ (g l^−1^)	10.4	22	38
Fe (mg l^−1^)	2.2	4.2	8
Tot_miner_ (g l^−1^)	28.1	54	80

*Temp, temperature; N_tot_, total nitrogen; C_tot_, total carbon; Tot_minerals_, Tot minerals. Detection limit of oxygen and sulfide is about 0.1 mg L^−1^*.

Both 16S rRNA amplicon pyrosequencing and metagenomic approaches have uncovered an unprecedented magnitude of microbial species, including previously unknown dominant microbes, and can provide effective assessments of microorganisms (Pedrós-Alió, 2012), community structure, and ecological importance of environmental viruses (Edwards and Rohwer, 2005) as well as gene contents; this thus enables the prediction of functional roles in various ecosystems such as the deep sea (Sogin et al., 2006), global oceans (Rusch et al., 2007), Amazon river (Ghai et al., 2011), acidic lake (Bendall et al., 2016), eutrophic lake (Garcia et al., 2018), meromictic lakes (Lauro et al., 2011; Comeau et al., 2012; Yau et al., 2013; Gies et al., 2014), underwater cave systems (Tetu et al., 2013), and among others.

Studies on marine viruses have demonstrated their contributions to the microbial community and the regulation of biogeochemical cycling (Suttle, 2007; Breitbart, 2012). Furthermore, the interaction between bacterioplankton populations and those of its phages have been uncovered in freshwater lakes and reservoir ecosystems (Roux et al., 2012; Skvortsov et al., 2016; Ghai et al., 2017), but still few studies have been employed on the ecologically important meromictic salt lakes. Meromictic salt lake ecosystems are known to have specific hydrochemical parameters and harbor particular microorganisms, providing excellent sedimentary archives of possible global paleoclimatic events (Dix et al., 1999), however they have received less attention than other aquatic ecosystems (Degermendzhy et al., 2010). Therefore, we chose to study Lake Shunet, a high-latitude meromictic salt lake in central Asia. The objectives of this study were to (1) explore the relationships between gene content or limnological parameters and microbial community structures over a depth profile; (2) reconstruct elemental nutrient cycles and investigate the potential role of the prevalent unclassified bacterial and archaeal groups in nutrient cycles; (3) unravel the history of potential interactions among viral, bacterial, and archaeal assemblages on the basis of the detection of clustered regularly interspaced short palindromic repeats (CRISPRs) and correlation of abundance profiles.

## Materials and methods

### Geographic description of study site and sampling procedures

Sampling was conducted in Lake Shunet (54° 25′N, 90° 13′E), which is 1.2 × 0.4 km in size (area, 0.47 km^2^; depth, 6.2 m). This saline meromictic lake is located in the northern polar region in the Republic of Khakassia, Siberia (Parnachev and Degermendzhy, 2002). It is permanently stratified, comprising an upper oxic layer (mixolimnion, 0.0–4.0 m), a transition layer (chemocline, 5.0 ± 0.2 m), and a lower anoxic layer (monimolimnion, 5.5–6.0 m) (Kallistova et al., 2006; Degermendzhy et al., 2010).

All samples were collected on July 21, 2010. Approximately 20 L of water was pumped from pre-defined depths corresponding to each stratified layer (i.e., 3.0, 5.0, and 5.5 m), and the water was then collected in sterile containers for transportation. The entire sampling time was ~2–2.5 h, including transportation of the samples back to the field station at Shira. The samples were filtered immediately at the station to reduce the potential bias in our samples involving transportation. These samples were subsequently filtered through a 10-μm plankton net and placed in a Millipore-Pellicon TFF system (0.22-μm filter membrane) to separate microbes and viruses (Tseng et al., 2013). Microbes were retained on 0.22-μm polycarbonate membrane filters, and viruses were collected from the filtrate water using a 50-kDa filter, which means viruses larger than 0.22 μm were excluded in the analysis. The viral samples were treated with DNase I (New England Biolabs UK Ltd., Herts, UK). The virus-like particles were purified in cesium chloride gradients by ultracentrifugation at a density of 1.35–1.50 g mL^−1^ (Angly et al., 2006; Tseng et al., 2013) for subsequent observation using TEM.

### Analysis of limnological parameters

The vertical profiles of temperature, dissolved oxygen (O_2_), and pH were measured using Hydrolab Data-Sonde 4a (Hydrolab, Austin, TX, U.S.A.) and YSI 6600 (Yellow Springs, OH, U.S.A.) submersible profilers. Conductivity readings at *in situ* temperatures (*C*_*t*_) were standardized to specific conductance at 25°C using the formula *K*_25_ = *C*_*t*_ × (1 + 0.0204 × (*T*-25)) ^−1^, where *T* is the *in situ* temperature in degrees Celsius (Hydrolab, YSI). Salinity was calculated on the basis of the relationship between salinity and conductivity, *S* (g L^−1^) = 1.117 *K*_25_ – 7.9716. The formula for conductivity and salinity (determined as ash content) was derived from water obtained from Lake Shira, another meromictic salt lake located 8 km northwest of Lake Shunet. Conductivity sensors were calibrated against 0.2 M KCl (Hydrolab, YSI) before each sample was collected. To determine sulfide concentrations, subsamples were fixed with zinc acetate and measured using the colorimetric method (Volkov and Zhabina, 1990). Total carbon (C_tot_) and total nitrogen (N_tot_) were determined by the CN–elemental analyzer FlashEA 1112 NC Soil/MAS 200 (Neolab LLC, U.S.A.). Ferric (Fe) and Sodium (Na) were detected using an inductively coupled argon plasma optical emission spectrometer. Phosphorus (P), ${\text{NH}}_{4}^{+}$, ${\text{NO}}_{2}^{-}$, ${\text{NO}}_{3}^{-}$, ${\text{SO}}_{4}^{2-}$, and total minerals (Tot_miner_) were all measured as described by Kalacheva et al. (2002). Limnological parameters were checked for collinearity using Spearman's rank correlation, and a set of non-collinear parameters was maintained for further analyses.

### DNA extraction, archaeal/bacterial amplicon libraries, and DNA sequencing

The membrane filter with microbial retentate was rinsed with sterile double-distilled water and subsequently with 10-mL Milli-Q water. The filter was then transferred into a 50-mL Falcon tube for microbial DNA extraction using the cetyltrimethylammonium bromide method (Wilson, 2001). Viral DNAs were extracted from purified virus-like particles using the formamide/cetyltrimethylammonium bromide method (Angly et al., 2006) and inspected using agarose gel electrophoresis. To ensure that sufficient viral DNA was available for sequencing, viral DNAs were amplified using a Genomiphi kit (GE Healthcare Life Science, Piscataway, NJ, U.S.A.).

A portion of microbial DNAs was used to analyze the bacterial and archaeal community composition on the basis of the V4 and V1-V2 hypervariable regions of the 16S rRNA gene. These amplicon libraries were generated using the universal primer pair of the 16S rRNA gene, namely 571F/751R for archaea and 27F/341R for bacteria. The PCR reaction was carried out in a total volume of 50 μL, containing 2.5U *Superrun EX taq*^TM^ HS, 5 μL of 10X *EX taq* Buffer, 200 μM of dNTPs, 0.2 μM of each primer, and 2–5 μg of diluted template DNA (final concentration 100 ng). The PCR program included an initial denaturation at 95°C for 5 min, followed by 30 cycles of 95°C for 20 s; 54°C for 10 s for archaea or 52°C for 20 s for bacteria; 72°C for 20 s; and finally 72°C for 5 min, with cooling at 4°C. Thereafter, PCR products were separated by 1% agarose gel electrophoresis with 1X TE Buffer and SYBR® Green I. Bands of expected sizes (~180 bp for archaea and 315 bp for bacteria) were cut from the gel and purified using a QIAEX II Gel Extraction Kit (QIAGEN Inc., Valencia, CA, U.S.A.). Purified DNA fragments were quantified using a NanoDrop spectrophotometer (Thermo Scientific, Vantaa, Finland). In the second round of the PCR process, individual tags were added to 5′ends of the 571F/751R and 27F/341R primers for each sample. The PCR mixture contained 2.5U *TaKaRa EX taq*^TM^ HS (TaKaRa Bio, Otsu, Japan), 5 μL of 10X *EX taq* Buffer, 200 μM of dNTPs, 0.4 μM of each tagged primer, and 100 ng V4/ V1-V2 amplicon in a final volume of 50 μL. The PCR program for tag addition had an initial denaturation at 95°C for 5 min, followed by five cycles of 95°C for 20 s, 56°C for 10 s, and 72°C for 20 s, with the final step of 72°C for 5 min, and then cooling at 10°C. The PCR products were purified, and a 200-ng mixture of tagged V4/V1-V2 regions was subject to 454 pyrosequencing using the Roche GS454 FLX Titanium System (Roche 454 Life Sciences, Branford, CT, U.S.A.) at Mission Biotech (Taipei, Taiwan).

After quality trimming of sequences, including chimera checking, and removal of ambiguous nucleotides (N), mismatched primers, and incomplete barcodes, 44,281/13,894 qualified bacterial/archaeal reads were retained. These reads were sorted into subgroups according to specific barcodes and then classified using a Ribosomal Database Project classifier (v2.3) with a bootstrap value of 0.8 for taxonomic assignment of sequences (Wang et al., 2007). Chloroplast reads were removed from subsequent analyses. Reads of each sample were aligned using MUSCLE (http://www.drive5.com/muscle). The distance matrix was calculated using PHYLIP package (v3.69) and clustered in MOTHUR (v.1.14.0) (http://www.mothur.org/) to assign OTUs at a threshold of 97% sequence similarity (Schloss et al., 2009). Notably, singletons were used only in estimating community richness and diversity; otherwise, they were excluded from analysis. In taxa names, NA means “not available at further levels.” Comparison studies of Lake Shunet with Sakinaw (Gies et al., 2014), Ursu, and Fara Fund lakes (Andrei et al., 2015) were executed using the same bioinformatics strategy described in Gies et al. (2014); for example, taxonomic assignments for both archaeal and bacterial sequences were blasted against the SILVA database.

### Sequencing, assembly, and annotation of metagenomes

The purified microbial and viral DNAs (i.e., metagenomes) were sequenced on an Illumina HiSeq 2000 sequencing platform (San Diego, CA, U.S.A.) at Yourgene Bioscience (Taipei, Taiwan). Metagenomic reads with ambiguous nucleotides (>2) and short lengths (<35 bp) were removed and assembled through a *de novo* assembly algorithm within the CLC Genomics Workbench using a 40-bp minimum overlap and 99% consensus. ORFs and 16S rRNA were predicted on assembled contigs using MetaGeneMark (http://exon.gatech.edu) and RNAmmer (v1.2) (Lagesen et al., 2007). Taxonomic assignment of predicted 16S rRNA was executed using the Ribosomal Database Project classifier (v2.3) with a bootstrap value of 0.8. ORFs were annotated by searching against the EggNOG (http://eggnog.embl.de) and Kyoto Encyclopedia of Genes and Genomes protein (Kanehisa et al., 2012) databases using BLASTp (*e*-value ≤ 10^−5^ and bit score ≥ 100). The abundance of ORFs was initially calculated on the basis of the coverage of read counts and then normalized by total sample size as relative abundance for further analyses. Data were log(x+1) transformed, and COG functional annotation was evaluated using STAMP (Parks and Beiko, 2010).

### Comparative metagenomic analyses

For comparative analysis, metagenomic reads of Ace Lake (filtered by 0.8 and 3.0 μm) were assembled using the same assembly workflow (https://www.imicrobe.us/) with default settings. ORF prediction and functional annotation were then performed using the same strategy applied to the Lake Shunet metagenomes (see *Sequencing, assembly, and annotation of metagenomes*). The ORF abundance was normalized by total sample size as relative abundance for the analysis. NMDS with Bray–Curtis distance metrics was used to explore the similarity in COG profiles between Ace Lake and Lake Shunet. Statistical analyses were performed using the Vegan package (Oksanen et al., 2011) in R (https://www.r-project.org/).

### Metagenomic binning and reconstruction of carbon, nitrogen, and sulfur cycles

Metagenomic contigs (>1,000 bp) were binned using a two-tiered binning algorithm (Saeed et al., 2012). The binning method used has previously been validated on both simulated and real-world data, similar to the data sets explored in this study (Saeed et al., 2012). Each bin was subjected to a conservative BLAST/MEGAN analysis to confirm that bin was meaningful. The binning method takes advantage of unsupervised machine learning to group related sequences into phylogenetic clusters (a technique which relies less on training data, which can skew results, and more on extracting common patterns in the data itself). The two-tiered nature of the algorithm (a course grouping, followed by a more refined grouping) was demonstrated to group sequences at a higher resolution and with better accuracy than other comparative methods. Moreover, the unsupervised characteristics of the binning method make it highly aligned with metagenomic studies, such as this one, where a complete set of *a priori* information about the underlying communities is not always available. Therefore, the selected binning method was an ideal candidate to aid the analysis of raw sequence data, and that the results obtained using the binning method are representative of the underlying microbial communities that we expected to find. However, it is worthy to note that the taxonomy assignment result would have some variation using different binning methods. ORFs were predicted using the contigs contained within each microbial bin, and flavobacteria from the viral samples were annotated using a BLASTp search against the KEGG protein database (*e*-value ≤ 10^−5^) to reconstruct carbon (C), nitrogen (N), and sulfur (S) cycles in Lake Shunet. The relative abundance of each bin was initially calculated according to the read counts of coverage of ORFs and subsequently normalized by total sample size.

### Bioinformatics analyses of viral communities and measurement of diameter of virus-like particles

Viral richness and the Shannon–Wiener Index (*H*′) were estimated using PHACCS (v1.1.3, http://sourceforce.net/projects/phaccs), on the basis of contig spectra generated by Circonspect (v0.2.5, http://sourceforge.net/projects/circonspect) in combination with the Cap3 assembler. Viral community composition was defined according to a homology search. Viral ORFs were searched against the NCBI RefSeq viral and microbial protein collection (ftp://ftp.ncbi.nlm.nih.gov/refseq/release) using BLASTp (*e*-value ≤ 10^−5^, single best match) to infer taxonomic information. Only 1.06, 0.88, and 0.82% of ORFs in the respective viral metagenomes were assigned to viruses (Figure S1). The final taxonomy of each contig was determined through the voting of constituted ORFs. However, many viral ORFs were not found to have any corresponding sequences in the databases; the composition prediction might be biased due to limited information. The relative abundance of each viral taxon was measured as described by Tseng et al. (2013). The function of viral ORFs was annotated using GhostKOALA of the KEGG database.

The diameter of virus-like particles was measured on the basis of TEM images using NIS-Elements AR 4.0 of Nikon microscopy and was calculated as follows:

$$\begin{array}{lll} \text{Diameter of virus}-\text{like particle} & = & \frac{\text{nm }\left(\text{scale bar}\right)}{\text{pixels }\left(\text{scale bar}\right)}\\ \times \text{ pixels of virus}-\text{like particle} \end{array}$$

### Analysis of flavobacterial genomes in viral samples

Several contigs derived from viral metagenomes (the largest being ~4.1 Mb) had hits against flavobacteria with a cutoff *e*-value of 10^−5^ and bit score of ≥100. Analyses involving 16S rRNA genes, reciprocal BLAST, and genome completeness confirmed that these contigs belonged to flavobacteria. Furthermore, a reciprocal BLASTp was used to examine the similarity of flavobacteria from microbial and viral samples, with a cutoff *e*-value of 10^−5^ and bit score of ≥100. Hidden Markov models using TIGRFAM/Pfam libraries (Haft et al., 2003; Finn et al., 2010) and CheckM (Parks et al., 2015) were used to estimate genome completeness. The fragment recruitment analysis was conducted using MUMmer (http://mummer.ourceforge.net/manual/) with default settings. Moreover, rRNA genes were predicted with WebMGA (Huang et al., 2009), and BLASTn searches against the NCBI nucleotide database (nt) were performed to identify taxonomically related species. According to BLASTn results, *Nonlabens dokdonensis* DSW-6 was the best hit (97% similarity), followed by *Psychroflexus torquis* ATCC 700755 (92% similarity) and Flavobacteria bacterium BBFL7 (92% similarity). The CLC Genomics Workbench (CLC bio) software was used to map contigs assigned to flavobacteria onto a draft genome. Contigs measuring >10 kb were used for single-copy gene analysis to estimate genome completeness. A genome map was drawn with Circos (Krzywinski et al., 2009) and annotated according to reference genomes (*Nonlabens dokdonensis* DSW-6, *Psychroflexus torquis* ATCC 700755, and Flavobacteria bacterium BBFL7).

### Analyses of interactions between microbial and viral assemblages

Appropriate computational approaches can predict bacteriophage–host relationships (Edwards et al., 2016). We used signal categories such as abundance profiles, CRISPRs, and exact matches. Congruence between ordinations of viral and bacterial/archaeal communities was quantified using the procrustean superimposition method (Procrustes) and examined for statistical significance using a Monte Carlo procedure (999 permutations). The *m*_12_ values, representing a goodness-of-fit statistic that measures congruence between two ordination configurations, were transformed to Procrustes correlation (r) by calculating the square root of their complements using the Vegan package (Oksanen et al., 2011). Pearson correlation coefficients between viral and bacterial/archaeal diversity indices and the richness estimate were calculated using the Psych package (Revelle, 2011). CRISPRs were predicted by PILER-CR (v1.06, http://www.drive5.com/pilercr) with default settings. To predict potential interactions between hosts and viruses, reciprocal BLAST analysis between microbial and viral metagenomics was initially conducted, followed by ORF clustering, using CD-HIT with a threshold of 90% sequence similarity (Li and Godzik, 2006). Moreover, contigs of microbial bins were searched against viral contigs to infer potential host–virus interactions (BLASTn, *e*-value ≤ 8 × 10^−21^, bits ≥ 100).

### Statistical analyses

The following statistical analyses were undertaken with R software (Team, 2011). Functional diversity was calculated using the R package ShotgunFunctionalizeR (Kristiansson et al., 2009). Count data (including community structure and functional profiles) were transformed into relative abundance by dividing by sample size. Limnological parameters were log(x+1) transformed for all analyses. The clustering heat map was generated using a gplots package (Bolker et al., 2012). Differences among the three depths in terms of contents of genes involved in energy metabolism were examined using the Wilcoxon test (Bauer, 1972), whereas Student's *t*-test was used for testing differences in viral diversity, richness estimates, and viral-like particle sizes. Non-metric Multidimensional Scaling (NMDS) based on Bray–Curtis distances was performed using the Vegan package to visualize the distribution of the bacterial/archaeal community composition or the 50 most abundant functional genes involved in metabolism, followed by *post-hoc* regression of individual explanatory variables on the ordination scores. Goodness-of-fit values and their significance were calculated using 999 random permutations. Correspondence analysis (Perrière and Thioulouse, 2003) using the Vegan package was applied to compare the variances of functional genes involved in energy metabolism and the 10 most abundant unclassified bacterial/archaeal OTUs. Only the top 50 genes based on the derived relative abundance were shown on the ordination plot.

### Phylogenetic analysis

Phylogenetic trees of the functional gene *psbA* were constructed using the neighbor-joining method from 500 bootstrap iterations using MEGA 6.0 (Tamura et al., 2013). In addition to the four partial *psbA* genes obtained from virome data in this study, we downloaded genes of various *Synechococcus* and *Prochlorococcus* phages from the UniProt and NCBI databases. Phylogenetic trees were constructed separately, because of the lack of common regions among the four partial *psbA* genes.

## Results

### Physicochemical profile of lake water

The gradient of each physicochemical parameter varied according to depth (Table 1). Both pH (8.1–6.7) and temperature (15.5–7.5°C) gradually decreased with depth. Furthermore, O_2_, H_2_S, and salinity changed sharply with depth; specifically, the O_2_ level was reduced from 10.9 mg L^−1^ to nearly zero in the chemocline, whereas the salinity (~71 g L^−1^) and H_2_S (~400 mg L^−1^) levels were much higher in the monimolimnion (i.e., bottom water).

### Specific bacterial and archaeal community structure and high diversity along the depths in lake shunet

Three representative depths for the mixolimnion (3.0 m), chemocline (5.0 m), and monimolimnion (5.5 m) layers were selected for a microbial community survey, according to hydrographical data. Archaeal and bacterial 16S ribosomal RNA genes were subjected to massively parallel pyrosequencing to characterize the community composition. After tagging and quality trimming of sequences, qualified sequences were submitted to the RDA pipeline to define the operational taxonomic unit (OTU) at 97% nucleotide identity. Following the removal of singletons, a total of 293 archaeal and 125 bacterial OTUs were identified. According to Shannon's diversity index (*H*′), the 5.5- and 3.0-m depths had the most diverse bacteria (*H*′ = 5.46) and archaea (*H*′ = 6.24), respectively. Rarefaction curves showed that only the curves for samples obtained at the 5.0-m depth approached an asymptote, suggesting that additional sequencing efforts at the other two depths would detect greater diversity.

**Figure 1:**
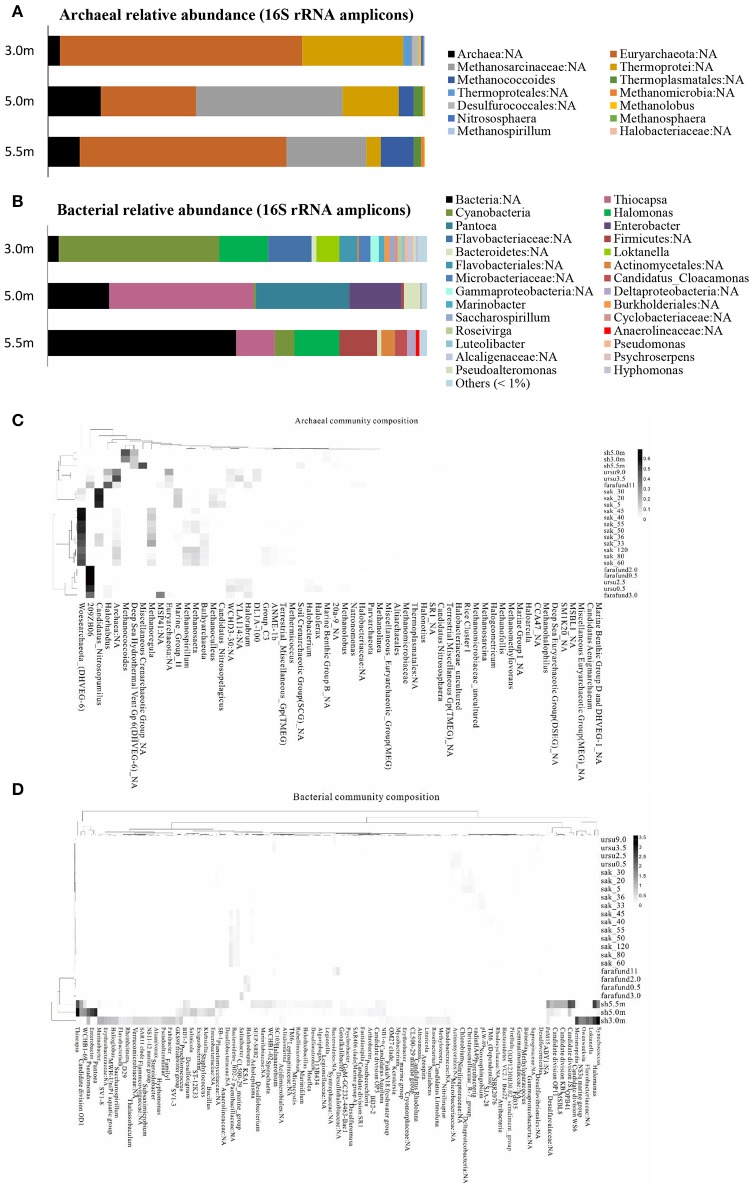
Community structure of **(A)** archaea and **(B)** bacteria based on 16S rRNA amplicons (>1% relative abundance) at the genus level across the three depths of Lake Shunet. Heat map and functional clustering based on relative abundance (>1%) of **(C)** archaeal and **(D)** bacterial assemblages (against SILVIA database) of meromictic Shunet, Sakinaw, Ursu, and Fara Fund lakes along the depths at the genus level. sh, Lake Shunet; sak, Sakinaw Lake; ursu, Ursu Lake; farafund, Fara Fund Lake. Bacterial community compositions of the four meromictic lakes respectively cluster while Lake Shunet and Sakinaw Lake harbored common archaeal groups.

The archaeal and bacterial community structures comprised 3 phyla, 5 classes, and 5 genera and 9 phyla, 15 classes, and 26 genera (relative abundance > 1%), respectively, with the corresponding prevailing phyla being *Euryarchaeota* and *Proteobacteria*. Most archaeal sequences were affiliated with a *Euryarchaeota* in both the upper and lower layers and with *Methanomicrobia* in the chemocline. The bacterial assemblage of the upper layer comprised pre-dominantly *Cyanobacteria*, whereas *Gammaproteobacteria* were abundant in the other lower layers. At the genus level, an unknown genus of *Euryarchaeota* and *Thiocapsa* were the most abundant archaea and bacteria, respectively (Figures 1A,B). Every depth harbored unique archaea and bacteria: *Nitrososphaera* and *Methanospirillum* were only present specifically in the mixolimnion (Figure 1A), and specific depth-dependent bacterial genera were also identified, including *Loktanella* in the mixolimnion, *Enterobacter* in the chemocline, and Candidatus *Cloacamonas* in the monimolimnion (Figure 1B). Notably, pre-dominant unclassified bacteria and archaea, as well as those at unknown genus levels, were observed across the three depths (Figures 1A,B), which echoed the relative abundance of genomic bins, revealing that unknown bacteria were abundant specifically in both upper and lower layers (Table 2).

**Table 2:** Summary of the results of the two-tiered binning approach applied to microbial metagenomes.

**Depth**	**Bin**	**Length (bp)**	**GC (%)**	**Assignment (BLAST/MEGAN)**	**Read counts**	**Relative abundance (%)^a^**	**Completeness (%)**	**Relative abundance (%)^b^**
3.0 m	3-1	5,320,493	42.62	*Bacteroidetes* (with hits to *Cyclobacterium marinum* DSM 745)	3,854,311	10.7	75.9	
	3-2	4,794,946	38.62	*Flavobacteria*	5,655,471	15.7	96.6	
	3-3	1,367,999	40.41	*Pseudoalteromonas*	626,217	1.7	16.4	
	3-4	1,817,139	49.62	Unknown bacterium	1,282,507	3.6	28.5	
	3-5	320,919	48.99	*Gammaproteobacteria*/*Rheinheimera*	94,248	0.3	12.1	
	3-6	2,895,826	57.63	Unknown bacterium	3,566,847	9.9	45.7	
	3-7	616,028	57.66	*Pseudomonas stutzeri*-like bacteria	1,061,784	3.0	15.5	9.6
	3-8	3,571,362	56.35	*Gammaproteobacteria*	4,187,146	11.7	61.2	
	3-9	498,520	57.30	*Halomonas*	1,665,901	4.6	7.1	
	*3-10*	*829,727*	65.51	*Rhodobacteraceae*	747,233	2.1	20.8	
	*3-11*	*991,406*	64.10	*Hyphomonas neptunium*-like bacteria	1,326,452	3.7	29.3	
	3-12	591,555	66.11	*Chroococcales*	8,087,523	22.5	10.3	
	3-13	1,175,729	65.17	*Gammaproteobacteria*/*Alcanivorax*	1,584,184	4.4	35.3	
	3-14	506,831	63.41	*Verrucomicrobia*/*Chthoniobacter flavus*	776,219	2.2	20.0	
5.0 m	5-1	1,745,731	34.09	*Firmicutes*/*Clostridia*	898,099	0.5	78.4	
	5-2	207,265	33.78	*Staphylococcus*	41,045	0.0	12.9	
	5-3	1,149,980	44.71	*Bacteroidetes* (with hits to *Anaerophaga thermohalophila* DSM 12881)	1,007,171	0.6	22.0	
	5-4	1,769,280	43.68	*Bacteroidetes* (with hits to *Anaerophaga thermohalophila* DSM 12881)	631,419	0.4	50.8	26.1
	5-5	4,105,682	55.09	*Enterobacteriaceae*	5,182,125	3.0	69.0	
	5-6	1,626,616	64.30	*Thiocapsa*	36,708,399	21.6	26.7	
5.5 m	5.5-1	233,710	28.74	Uncultured bacterium (with hits to *Halanaerobiales*)	260,650	0.1	13.9	
	5.5-2	1,215,938	31.03	Uncultured bacterium (with hits to *Halanaerobium*)	1,595,420	0.6	62.4	
	5.5-3	1,005,967	35.40	Uncultured bacterium (with hits to *Bacteroidetes*, *Firmicutes* and *Proteobacteria*)	990,730	0.4	52.6	
	5.5-4	451,924	35.33	*Halanaerobium*	344,630	0.1	21.2	
	5.5-5	435,003	36.34	Uncultured bacterium (with hits to *Firmicutes*)	235,873	0.1	48.6	
	5.5-6	1,084,706	37.25	Uncultured bacterium (Candidatus *Cloacamonas acidaminovorans* str. Evry)	4,584,399	1.7	50.7	
	5.5-7	503,708	39.05	Uncultured *Desulfobacterium* sp. (with hits to delta proteobacterium NaphS2)	769,643	0.3	8.6	
	5.5-8	361,955	38.51	*Bacteroidales*/*Marinilabiaceae*	216,055	0.1	12.1	10.8
	5.5-9	1,017,801	44.56	*Deltaproteobacteria*	667,527	0.3	30.1	
	5.5-10	1,029,185	47.56	Uncultured candidate division OP1 bacterium	4,071,850	1.5	42.3	
	5.5-11	605,053	45.43	*Sphaerochaeta pleomorpha*-like bacteria	490,201	0.2	33.3	
	5.5-12	1,051,753	47.58	*Desulfobacteraceae*	1,227,189	0.5	33.7	
	5.5-13	1,216,430	47.17	*Bacteroidetes*/*Marinilabiaceae*	674,195	0.3	37.9	
	5.5-14	271,285	53.82	Unknown bacterium	600,060	0.2	12.5	
	5.5-15	300,891	53.73	*Desulfobacteraceae*	249,040	0.1	12.0	
	5.5-16	419,569	54.53	*Clostridiaceae*	310,830	0.1	8.3	
	5.5-17	1,317,006	56.70	*Halomonas*	1,909,053	0.7	20.0	
	5.5-18	1,692,070	64.65	*Thiocapsa*	9,588,717	3.6	27.6	

*Taxonomic assignments were predicted according to BLASTp results (e-value < 10^−5^) using MEGAN, with a minimum support of 20 and a minimum bit score of 100 in alignment*.

a *Relative abundance was calculated according to the read counts of the bin divided by the total read counts*.

b *Relative abundance was calculated according to the total read counts of all bins per depth divided by the total read counts*.

Bacterial assemblages were dominated by *Cyanobacteria* in the mixolimnion and *Gammaproteobacteria* in both the chemocline and monimolimnion in Lake Shunet. Shunet differs from Sakinaw Lake, in which *Actinobacteria, Gammaproteobacteria*, and *Chloroflexi* are pre-dominant along the layers (Gies et al., 2014); Ursu and Fara Fund Lakes, in which *Gammaproteobacteria* or *Deltaproteobacteria* pre-dominate along these layers (Andrei et al., 2015). The bacterial community compositions of these four meromictic lakes, each have their own dominant genera. Lake Shunet, for example, has dominant bacteria that the other lakes do not, including *Thiocapsa, Candidate division OD1*, and *Halomonas* (Figure 1C). In terms of archaeal compositions, Lake Shunet and Sakinaw Lake were more similar to each other than the Ursu and Fara Fund Lakes; both groups of lakes harbored common archaeal groups (Figure 1D). These results reflect the stratified nature and uniqueness of meromictic lakes.

### Functional profiles varied among depths

Using a deep sequencing strategy to uncover and comprehend potential metabolisms of microbial communities in Lake Shunet, we generated sequences with sizes ranging between 26 and 72 Gb for each of the six samples (Table S1), as well as microbial and viral sequences with total approximate sizes of 132 and 133 Gb, respectively. Consequently, total bases of contigs were between 121 Mbp and 14 Gbp in length for the six metagenomes. The largest microbial and viral contigs were 0.47 and 0.09 Mbp, respectively (Table S1). A total of 1,302,473 microbial and 1,379,639 viral open reading frames (ORFs) were predicted from the contigs and were annotated in 4356 COGs families, of which 71.9% were shared by the three depths, and 9.1, 3.7, and 2.5% were specific to the respective depths. A functional diversity index was calculated on the basis of metagenomic data by using ShotgunFunctionalize R; the index of Lake Shunet (Shannon index = 7.67–7.81) was higher than that of the North Pacific Ocean (Shannon index = 6.46–6.93) (DeLong et al., 2006; Kristiansson et al., 2009).

**Figure 2:**
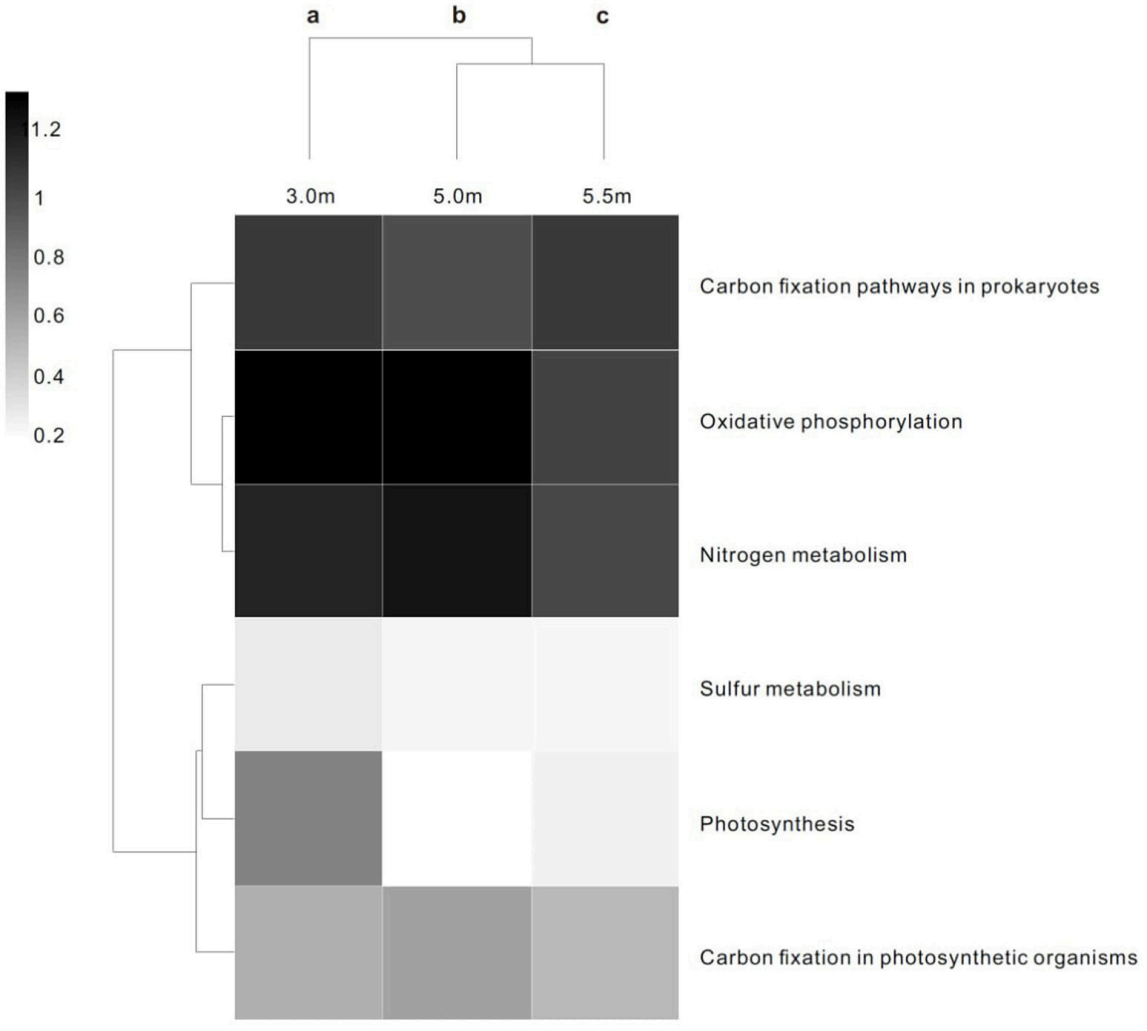
Heat map and functional clustering of predicted ORFs from microbial metagenomic contigs based on KO of energy metabolism at three depths. The heat scale is the relative abundance of ORFs assigned to each individual KO of energy metabolic category. ^a−c^Different letters denote a significant difference in the statistical test (*P* < 0.05).

The three layers harbored significantly different contents of genes involved in energy metabolism (*P* < 0.05; Figure 2). In terms of COG functional profiles, most of the genes were annotated as playing roles in replication, recombination and repair, amino acid transport and metabolism, cell wall/membrane/envelope biogenesis, and energy production and conversion. STAMP analysis conducted on the basis of the COG profiles revealed that the observed microbial metabolism, including energy production and conversion, significantly differed (*P* < 0.05) among the three depths (Figure S2).

### Relationships between metabolic functions and archaeal/bacterial community structure and limnological parameters

**Figure 3:**
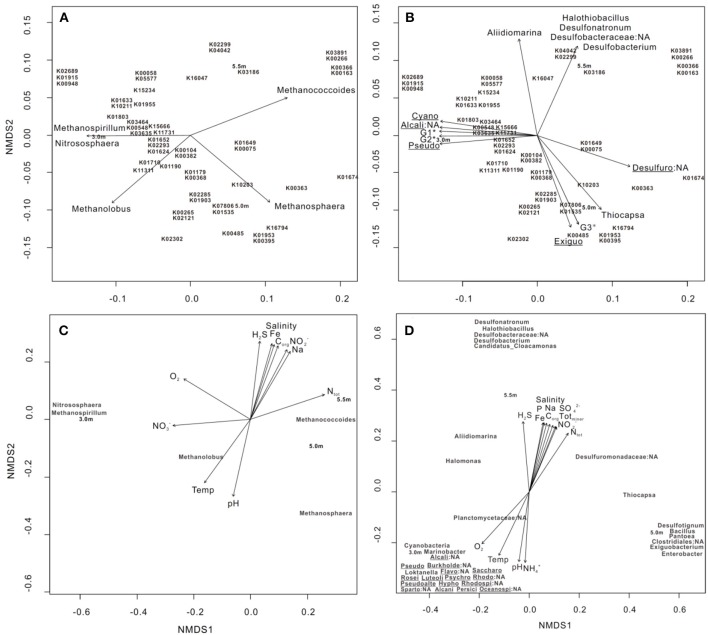
NMDS ordination of three sampling depths based on **(A,B)** the 50 most abundant KO involved in metabolic pathways, **(C)** bacterial and **(D)** archaeal community structures at the genus level (>0.1% relative abundance). In each diagram, archaeal, bacterial groups, and limnological parameters with a significant goodness of fit based on *post-hoc* correlations (*P* ≤ 0.05) are represented as vectors. In **(B)**, G1* includes Planctomycetaceae:NA and Marinobacter; G2* includes Spartobacteria:NA, Roseivirga, Loktanella, Rhodobacteraceae:NA, Oceanospirillales:NA, Psychroserpens, Luteolibacter, Burkholderiales:NA, Pseudoalteromonas, Persicivirga, and Alcanivorax and Flavobacteriales:NA; and G3* includes Pantoea, Desulfotignum, Bacillus, and Clostridiales:NA. Cyano: Cyanobacteria, Alcali: Alcaligenaceae, Pseudo: Pseudomonas, Desulfuro: Desulfuromonadaceae, Exiguo: Exiguobacterium, Burkholde: Burkholderiales, Flavo: Flavobacteriales, Saccharo: Saccharospirillum, Rosei: Roseivirga, Luteoli: Luteolibacter, Psychro: Psychroserpens, Rhodo: Rhodospirillaceae, Pseudoalte: Pseudoalteromonas, Hypho: Hyphomonas, Rhodospi: Rhodospirillaceae:NA, Sparto: Spartobacteria, Alcani: Alcanivorax, Persici: Persicivirga, and Oceanospi: Oceanospirillales. The 50 genes are outlined as follows: **K01633** dihydroneopterin aldolase, **K10211** 4,4′-diaponeurosporenoate glycosyltransferase, **K03186** 3-octaprenyl-4-hydroxybenzoate carboxy-lyase UbiX, **K01915** glutamine synthetase, **K00948** ribose-phosphate pyrophosphokinase, **K02689** photosystem I P700 chlorophyll a apoprotein A1, **K00265** glutamate synthase (NADPH/NADH) large chain, **K02121** V-type H+-transporting ATPase subunit E, **K00548** methyltetrahydrofolate– homocysteine methyltransferase, **K01674** carbonic anhydrase, **K01955** carbamoyl-phosphate synthase large subunit, **K01652** acetolactate synthase I/II/III large subunit, **K01624** fructose-bisphosphate aldolase, class II, **K02293** 15-cis-phytoene desaturase, **K02302** uroporphyrin-III C-methyltransferase/precorrin-2 dehydrogenase/sirohydrochlorin ferrochelatase, **K04042** bifunctional UDP-N-acetylglucosamine pyrophosphorylase/Glucosamine-1-phosphate N-acetyltransferase, **K02299** cytochrome o ubiquinol oxidase subunit III, **K00266** glutamate synthase (NADPH/NADH) small chain, **K03891** ubiquinol-cytochrome c reductase cytochrome b subunit, **K15666** fengycin family lipopeptide synthetase C, **K03464** muconolactone D-isomerase, **K00382** dihydrolipoamide dehydrogenase, **K00104** glycolate oxidase, **K01190** beta-galactosidase, **K01649** 2-isopropylmalate synthase, **K00075** UDP-N-acetylmuramate dehydrogenase. **K16794** platelet-activating factor acetylhydrolase IB subunit alpha, **K01803** triosephosphate isomerase (TIM), **K07806** UDP-4-amino-4- deoxy-L-arabinose-oxoglutarate aminotransferase, **K01535** H+-transporting ATPase, **K01953** asparagine synthase (glutamine-hydrolysing), **K00395** adenylylsulfate reductase, subunit B, **K01903** succinyl-CoA synthetase beta subunit, **K02285** phycocyanin beta chain, **K00058** D-3-phosphoglycerate dehydrogenase, **K05577** NAD(P)H-quinone oxidoreductase subunit 5, **K01710** dTDP-glucose 4,6-dehydratase, **K00363** nitrite reductase (NAD(P)H) small subunit, **K03635** molybdopterin synthase catalytic subunit, **K11731** citronellyl-CoA dehydrogenase, **K00485** dimethylaniline monooxygenase (N-oxide forming), **K10203** elongation of very long chain fatty acids protein 6, **K16047** 3-hydroxy-9,10-secoandrosta-1,3,5(10)-triene-9,17-dione monooxygenase subunit HsaA, **K00163** pyruvate dehydrogenase E1 component, **K00366** ferredoxin-nitrite reductase, **K15234** citryl-CoA lyase, **K11311** anthranilate dioxygenase reductase, **K01179** endoglucanase, **K00368**; nitrite reductase (NO-forming), and **K15655** surfactin family lipopeptide synthetase B.

Nonmetric multidimensional scaling (NMDS), based on Bray–Curtis distances with *post-hoc* regression, was used to explore the correlation between the 50 most abundant genes involved in KEGG orthology (KO) profiles of metabolism and bacterial/archaeal assemblages (>0.1% relative abundance). Five archaeal groups, including *Methanococcoides, Methanosphaera*, and *Methanolobus*, and bacterial groups, including *Desulfobacterium, Desulfuromonadaceae*, and *Thiocapsa*, had a significant goodness of fit (*P* ≤ 0.05) regarding the relative importance of the 50 most abundant genes involved in metabolism (Figures 3A,B). Specifically, *Methanococcoides* were correlated with K00163 (pyruvate dehydrogenase E1 component), K00366 (ferredoxin-nitrite reductase), K00266 (glutamate synthase (NADPH/NADH) small chain), and K03891 (ubiquinol-cytochrome c reductase cytochrome b subunit), whereas K10203 (elongation of very long chain fatty acids protein 6) was enriched by *Thiocapsa*.

We conducted NMDS on the basis of bacterial and archaeal community structures, followed by *post-hoc* regression of individual environmental variables; the results revealed that parameters including H_2_S, O_2_, ${\text{NH}}_{4}^{+}$, and ${\text{NO}}_{2}^{-}$, as well as 11 other parameters, significantly explained the variance of both community structures with depth (Figures 3C,D). Specifically, *Methanococcoides* and *Methanolobus* were positively associated with increases in total nitrogen (N_tot_) and temperature, respectively (Figure 3C). The bacterial clusters including *Desulfonatronum, Halothiobacillus, Desulfobacteraceae, Desulfobacterium*, and Candidatus_*Cloacamonas* were enriched as H_2_S increased, whereas *Cyanobacteria, Marinobacter, Alcaligenaceae*:NA, and *Planctomycetaceae*:NA were correlated with changes in O_2_ (Figure 3D).

### Reconstruction of nutrient cycles through the depths

**Figure 4:**
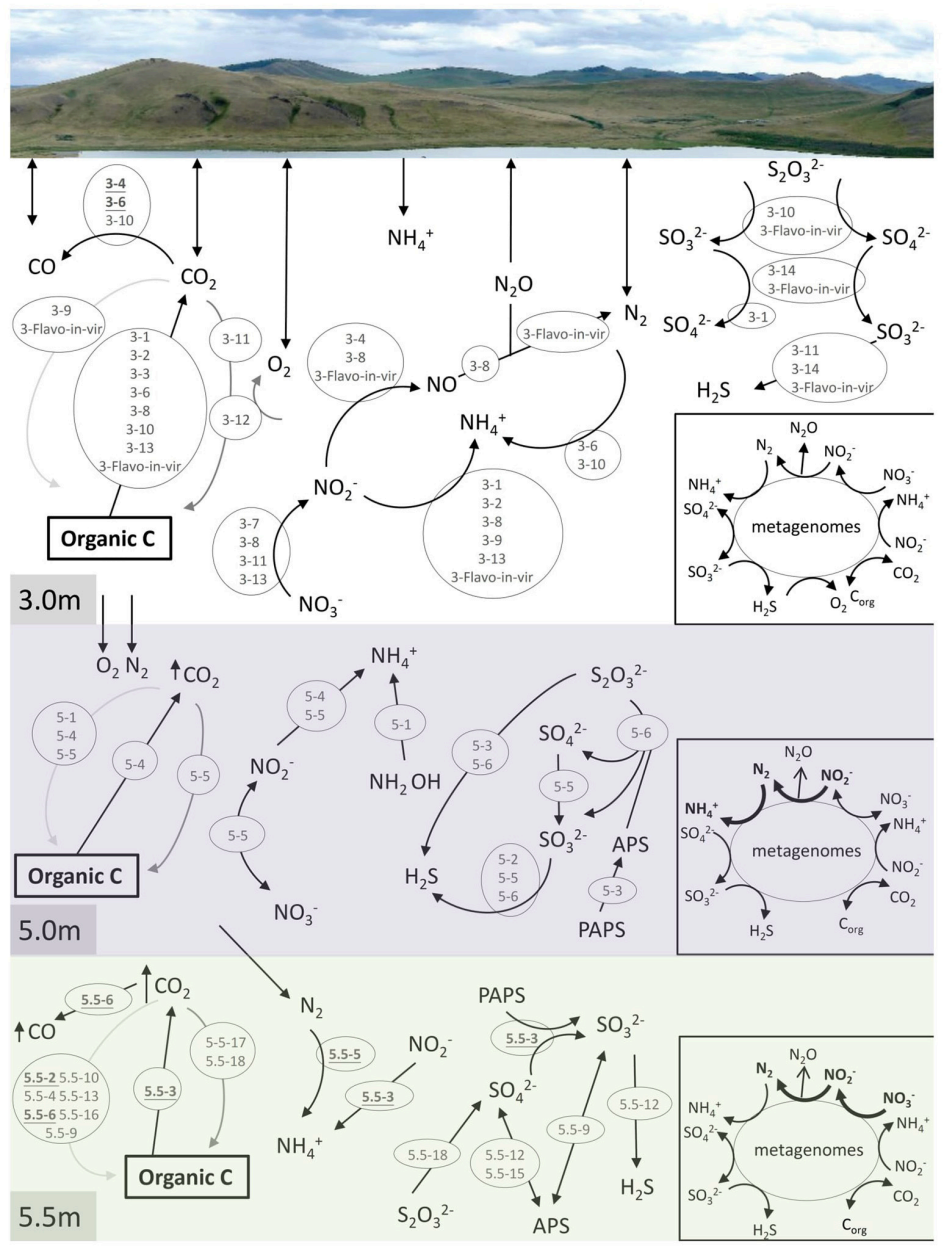
Reconstruction of C, N, and S cycles in Lake Shunet. The energy flux is presented for each depth based on the KEGG annotation of bins and metagenomes (inlet square at right-bottom corner). Numbers inside the gray circles denote bins. Arrows in bold indicate the presence of the pathways in the corresponding metagenomes, but not in bins. In the C cycle, black arrow: respiration, dark gray arrow: aerobic carbon fixation, and light gray arrow: anaerobic carbon fixation. PAPS: 3′-Phosphoadenylyl sulfate, PAS: Adenylyl sulfate, C_org_: Organic matter. Underlined numbers in bold represent unknown bacteria. 3-1: *Bacteroidetes*, 3-2: *Flavobacteria*, 3-3: *Pseudoalteromonas*, 3-4: unknown bacterium, 3-6: unknown bacterium, 3-7: *Pseudomonas stutzeri*-like bacteria, 3-8: *Gammaproteobacteria*, 3-9: *Halomonas*, 3-10: *Rhodobacteraceae*, 3-11: *Hyphomonas neptunium*-like bacteria, 3-12: *Chroococcales*, 3-13: *Alcanivorax*, 3-14: *Verrucomicrobia*/*Chthoniobacter flavus*, 3-Flavo-in-vir: *Nonlabens* sp. *sh3vir*., 5-1: *Firmicutes*/*Clostridia*, 5-2: *Staphylococcus*, 5-3: *Bacteroidetes*, 5-4: *Bacteroidetes*, 5-5: *Enterobacteriaceae*, 5-6: *Thiocapsa*, 5.5-2: Uncultured bacterium, 5.5-3: Uncultured bacterium, 5.5-4: *Halanaerobium*, 5.5-5: Uncultured bacterium, 5.5-6: Uncultured bacterium, 5.5-9: *Deltaproteobacteria*, 5.5-10: Uncultured candidate division OP1 bacterium, 5.5-12: *Desulfobacteraceae*, 5.5-13: *Bacteroidetes*/*Marinilabiaceae*, 5.5-15: *Desulfobacteraceae*, 5.5-16: *Clostridiaceae*, 5.5-17: *Halomonas*, 5.5-18: *Thiocapsa*. Please refer to Table 2 for information regarding each bin.

Many long-sized contigs obtained by deep sequencing considerably facilitated binning metagenomic data, thus enabling the prediction of potential elemental nutrient networks among the microbial groups. A total of 14, 6, and 18 microbial bins (namely taxonomically related groups) were identified in the respective metagenomes of the three depths (Table 2). The elemental nutrient cycles of the three layers were reconstructed according to the gene annotation in individual microbial genomic bins (Figure 4). Serial numbers were assigned to each bin in the form of “depth-number,” as shown in the following descriptions.

Carbon cycling (respiration, aerobic, and anaerobic carbon fixation) was reconstructed according to carbon fixation pathways, including reductive citric acid, hydroxypropionate-hydroxybutyrate cycles, and the Wood–Ljungdahl pathway. Groups of *Bacteroidetes* (3-1), *Flavobacteria* (3-2), *Pseudoalteromonas* (3-3), unknown bacteria (3-6), *Gammaproteobacteria* (3-8), *Rhodobacteraceae* (3-10), *Alcanivorax* (3-13), *Nonlabens* sp. *sh3vir*. (3-Flavo-in-vir), *Bacteroidetes* (5-4), and uncultured bacteria (5.5-3) carried genes encoding enzymes that oxidize organic carbon. Carbon fixation using oxygen as an electron acceptor was detected only in the bin of *Chroococcales* (3-12), the most abundant group in the mixolimnion. *Hyphomonas neptunium*-like bacteria (3-11), *Enterobacteriaceae* (5-5), and *Halomonas* (5.5-17) were non-phototrophic groups that harbored *prkB* or *rbcS* for autotrophic carbon fixation at the respective depths. Genes for anaerobic carbon fixation were identified in various microbes—including *Halomonas* (3-9), *Nonlabens* sp. *sh3vir*. (3-Flavo-in-vir), *Clostridia* (5-1), *Bacteroidetes* (5-4), *Enterobacteriaceae* (5-5), uncultured bacteria (5.5-2), *Halanaerobium* (5.5-4), uncultured bacteria (5.5-6), *Deltaproteobacteria* (5.5-9), uncultured candidate division OP1 bacteria (5.5-10), *Marinilabiaceae* (5.5-13), and *Clostridiaceae* (5.5-16)—at the three sampling depths, particularly in the anoxic monimolimnion. In addition, carbon monoxide (CO) was an intermediate in the Wood–Ljungdahl pathway of acetyl-CoA biosynthesis, possibly produced by the abundant unknown bacteria (3-4 and 3-6), *Rhodobacteraceae* (3-10), and the uncultured bacterium (5.5-6).

Nitrogen cycling, including denitrification, nitrogen fixation, and nitrate and nitrite reduction, was annotated at all sampling depths (Figure 4). In the samples derived at the 3.0-m depth, genes involved in denitrification for energy production were completely defined in the bins of *Pseudomonas stutzeri-*like bacteria (3-7), *Gammaproteobacteria* (3-8), *H. neptunium-*like bacteria (3-11), *Alcanivorax* (3-13), the unknown bacterium (3-4), and *Nonlabens* sp. *sh3vir*. (3-Flavo-in-vir). Additionally, *Bacteroidetes* (3-1), *Flavobacteria* (3-2), *Gammaproteobacteria* (3-8), *Halomonas* (3-9), *Alcanivorax* (3-13), and *Nonlabens* sp. *sh3vir*. (3-Flavo-in-vir) harbored genes for nitrite reduction. *P. stutzeri-*like bacteria (3-7), *Gammaproteobacteria* (3-8), *H. neptunium-*like bacteria (3-11), and *Alcanivorax* (3-13) were potentially capable of reducing nitrate. Moreover, nitrogenase (EC 1.18.6.1 that processes nitrogen fixation) was identified only in bins of the unknown bacterium (3-6) and *Rhodobacteraceae* (3-10). In the samples derived at the 5.0-m depth *Enterobacteriaceae* (5-5), constituting the second most abundant group after *Thiocapsa* in the chemocline, contained genes for both nitrate reduction and nitrite oxidation; by contrast, *Bacteroidetes* (5-4) contained genes involved in only nitrite reduction. *Clostridia* (5-1) contained genes involved in an alternative pathway to produce ammonium from hydroxylamine (NH_2_OH) in nitrogen cycling. In the samples derived at the 5.5-m depth, only uncultured bacteria (5.5-3 and 5.5-5) were detected to contain genes involved in the nitrogen cycle, mediating nitrite reduction and nitrogen fixation, respectively.

Genes involved in the key processes of sulfur cycling, including sulfite (${\text{SO}}_{3}^{2-}$), thiosulfate (S_2_${\text{O}}_{3}^{2-}$) oxidation, sulfate (${\text{SO}}_{4}^{2-}$) reduction, and sulfur disproportionation (S_2_${\text{O}}_{3}^{2-}$ 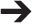
H_2_S + ${\text{SO}}_{4}^{2-}$), were detected at specific depths (Figure 4). Pathways for oxidizing thiosulfate to sulfate and sulfite were attributed to *Rhodobacteraceae* (3-10) and *Nonlabens* sp. *sh3vir*. (3-Flavo-in-vir) at 3.0 m and to *Thiocapsa* (5-6 and 5.5-18) at 5.0 and 5.5 m. Genes that encode sulfite oxidation were identified in bins including *Bacteroidetes* (3-1), *Deltaproteobacteria* (5.5-9), *Desulfobacteraceae* (5.5-12 and 5.5-15), *Chthoniobacter flavuz-like* bacteria (3-14), and *Nonlabens* sp. *sh3vir*. (3-Flavo-in-vir). Other bins such as *H. neptunium-*like bacteria (3-11), *Staphylococcus* (5-2), *Enterobacteriaceae* (5-5), *Thiocapsa* (5-6), uncultured bacteria (5.5-3), *Deltaproteobacteria* (5.5-9), and *Desulfobacteraceae* (5.5-12 and 5.5-15) could utilize sulfate as a terminal electron acceptor to produce H_2_S. *Bacteroidetes* (5-3) and uncultured bacteria (5.5-3) had genes to consume 3-phosphoadenylyl sulfate (PAPS), whereas *Thiocapsa* (5-6) could execute sulfur disproportionation to produce sulfate.

The constructed C, N, and S cycles annotated on the basis of the 3.0-m metagenome were also annotated in 3.0-m genomic bins; however, a few pathways involved in N cycles annotated on the basis of the 5.0 and 5.5-m metagenomes were not found in any genomic bins at the depths, such as nitrogen fixation, denitrification, and nitrate reduction. This means that some microbes playing a role in N cycles were still not defined.

### Virus-like particle size, viral genome size, diversity, and function annotation

**Figure 5:**
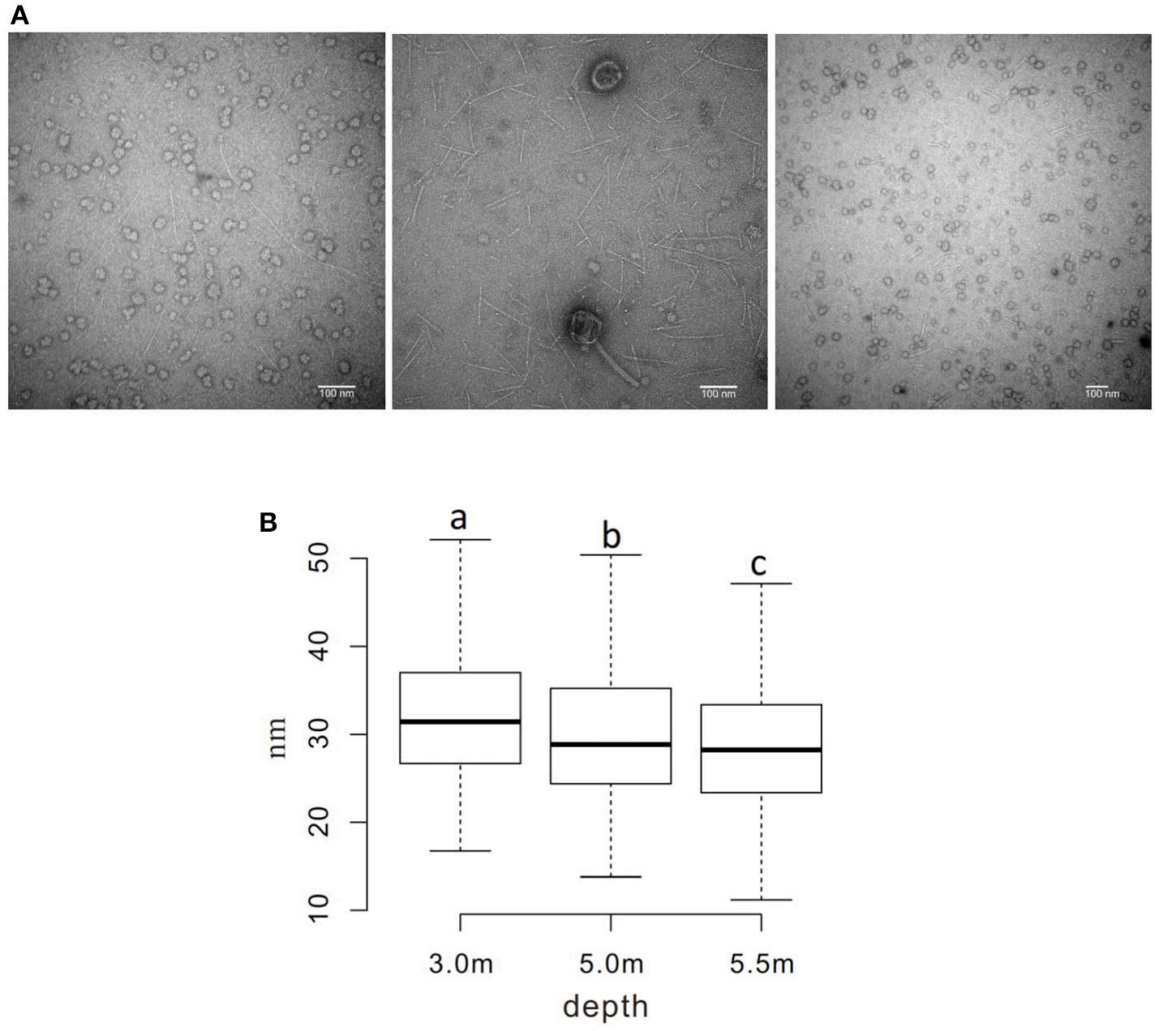
Measurement of the diameter of viral particles. **(A)** TEM photos of viral samples at each sampling depth (left to right: 3.0, 5.0, and 5.5 m) **(B)** Boxplot of measured diameters. ^a−c^Diameters without a common superscript differed (*P* < 0.05).

The sizes of virus-like particles were measured using transmission electron microscopy (TEM; Figure 5A); the results revealed that the sizes decreased significantly across the depths, with average sizes of ~ 32, 30, and 28 nm at the three sampling depths, respectively (*P* < 0.05; Figure 5B). A similar pattern was also observed in viral contig length, which was longer (28.9 kb on average) at 3.0 m than at 5.0 and 5.5 m (24.4 and 19.9 kbp, respectively).

**Figure 6:**
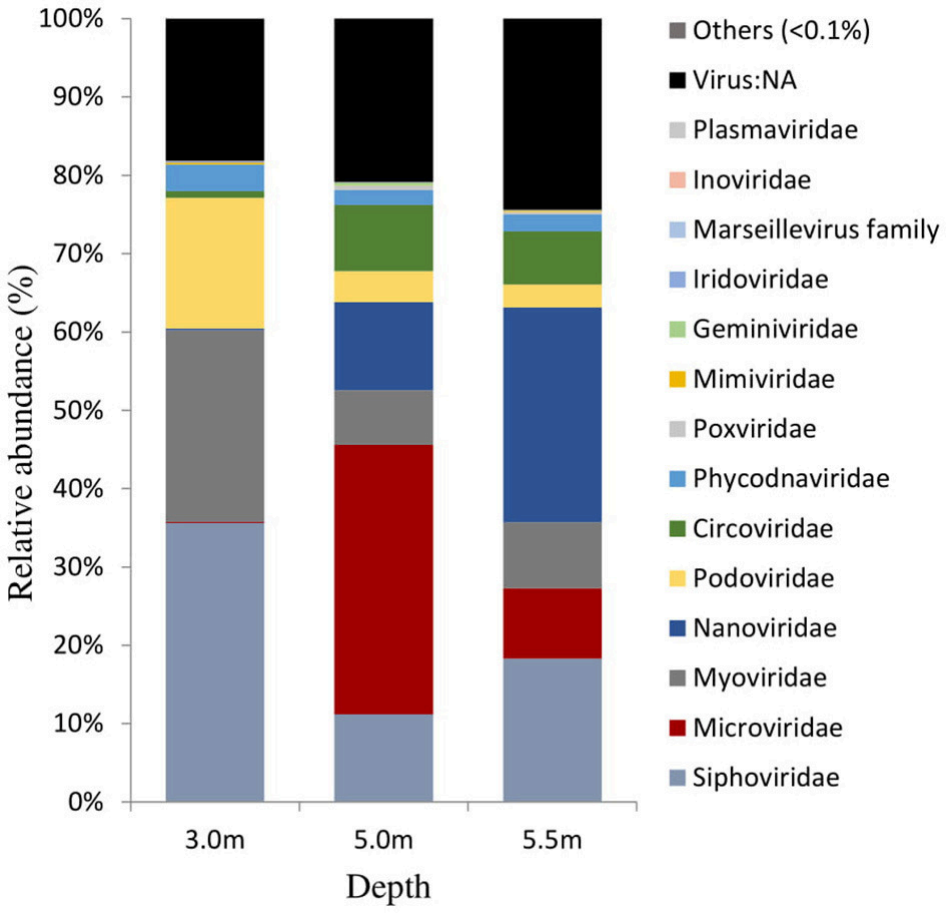
Viral community structures at the family level in Lake Shunet. Virus:NA represents unknown virus.

The viral community structure differed among the three sampling depths (Figure 6). Fourteen abundant viral families (>0.1% relative abundance) were detected through the water column, including several typical bacteriophage groups, such as *Siphoviridae, Microviridae, Myoviridae*, and *Podoviridae*, and three viral groups (*Nanoviridae, Circoviridae*, and *Phycodnaviridae*). The viral assemblage at 3.0 m was dominated by *Siphoviridae* (35.6%), followed by *Myoviridae*, whereas the viral assemblages at 5.0 and 5.5 m were dominated by *Microviridae* (34.4%) and *Nanoviridae* (27.4%), followed by *Nanoviridae* and *Siphoviridae*, respectively. Furthermore, many unknown viruses were detected at the three depths, with the greatest enrichment (24.4%) being observed at 5.5 m (Figure 6). Viral diversity determined using PHAACS differed significantly among the three depths (Table 3). The 3.0-m layer had the most genotype-rich (15,877 genotypes) and diverse (*H*′ = 9.15) viral assemblage, followed by the 5.5-m layer (10,000 genotypes and *H*′ = 8.06) and 5.0-m layer (2,498 genotypes and *H*′ = 6.70). The highest evenness estimate was derived at 3.0 m, indicating that all viral genotypes were close to being equally abundant, whereas some viral genotypes at 5.0 and 5.5 m were pre-dominant. The lowest evenness was observed in the viral community at 5.0 m, and the most abundant genotype constituted 5.51% of the community.

**Table 3:** Diversity and richness estimates from the viral metagenomes were determined using PHAACS.

	**3.0 m-V**	**5.0 m-V**	**5.5 m-V**
Best model	Power	Power	Power
Richness	15,877^a^	2,498^b^	10,000^c^
Evenness	0.94^a^	0.86^b^	0.87^c^
Most abundant genotype (%)	0.90	5.51	3.28
Shannon-Wiener Index (*H′*)	9.15^a^	6.70^b^	8.06^c^

a−c *Numbers without a common superscript differed (P < 0.05)*.

Viral ORFs, which pose as host-like metabolic genes, imply that a phage might have obtained genes from the host during a viral replication with or without purpose. In our data set, the viral ORFs were mostly annotated as playing roles in carbohydrate metabolism at 3.0 m and genetic information processing at both 5.0 and 5.5 m. Additionally, ~ 4.0, 3.0, and 2.0% of the viral ORFs were annotated as playing roles in energy metabolism, including key genes involved in nitrogen metabolism (*nirS, nifH*), sulfur metabolism (*dsrA, dsrB, sox, sir*), photosynthesis (*psaA, psaB, psbA, psbD*), and oxidative phosphorylation (*acs, ndh*), but not methane metabolism (*pmoA, mcrA*). Specifically, cyanophage-encoded auxiliary metabolic genes, including four partial *psbA* genes, were detected. In the phylogenetic analyses, only the gene_id_217671_5.0 m was clustered in an individual branch, close to D1_protein_Synechococcus_phage_S-SSM5 (Figure S3).

### Potential interactions among viral, archaeal, and bacterial assemblages

**Figure 7:**
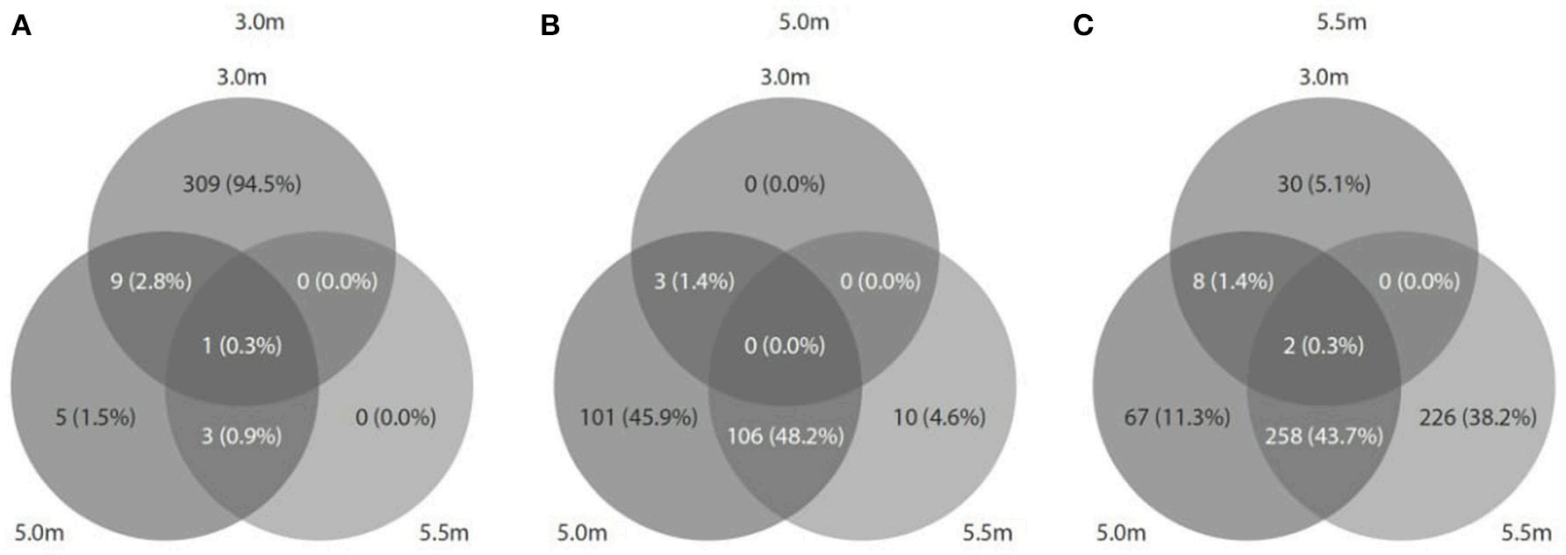
Predicted CRISPRs of microbial metagenomes were blasted against viral metagenomes at the corresponding depth. **(A)** At 3.0 m: 1094 CRISPRs (327 hits matched); **(B)** at 5.0 m: 1129 CRISPRs (220 hits matched), and **(C)** at 5.5 m: 2886 CRISPRs (591 hits matched).

Viral diversity was positively correlated with both bacterial (*r*^2^ = 0.558, *P* = 0.46) and archaeal diversity (*r*^2^ = 0.999, *P* < 0.05) and richness estimates (bacteria: *r*^2^ = 0.848, *P* = 0.25; archaea: *r*^2^ = 0.989, *P* = 0.06). Procrustes analyses revealed congruence among viral and bacterial (*m*_12_ = 0.99, *P* < 0.05) and archaeal assemblages (*m*_12_ = 0.99, *P* < 0.01). Approximately 1,094 (3.0 m), 1,129 (5.0 m), and 2,886 (5.5 m) CRISPR arrays were predicted from the respective microbial metagenomes, and most matches were observed between spacers and viral assemblages from the same depth (Figure 7). In addition, reciprocal BLAST analysis revealed ~ 74,777 (3.0 m), 3,761 (5.0 m), and 26,972 (5.5 m) hits between viral and microbial ORFs across the depths.

In total, 9, 22, and 13 unique viral sequences were found in microbial bin sequences at the respective depths (Table S2). The detected viral taxa, including *Cronobacter* phages Vb_CsaM_GAP31, *Pseudomonas* phage YuA, and *Prochlorococcus* phage P-SSM7, did not show host specificity to any bacteria. The microbial groups observed at the 3.0-m depth seemed to be attacked only by viruses obtained from this depth, except for *Flavobacteria* (3-2), which were attacked by *Cronobacter* phage obtained from the 5.0-m depth. However, the microbial groups observed at the 5.0- and 5.5-m depths seemed to be infected randomly by viruses from both depths.

### Flavobacteria in viral samples at 3.0 m

**Figure 8:**
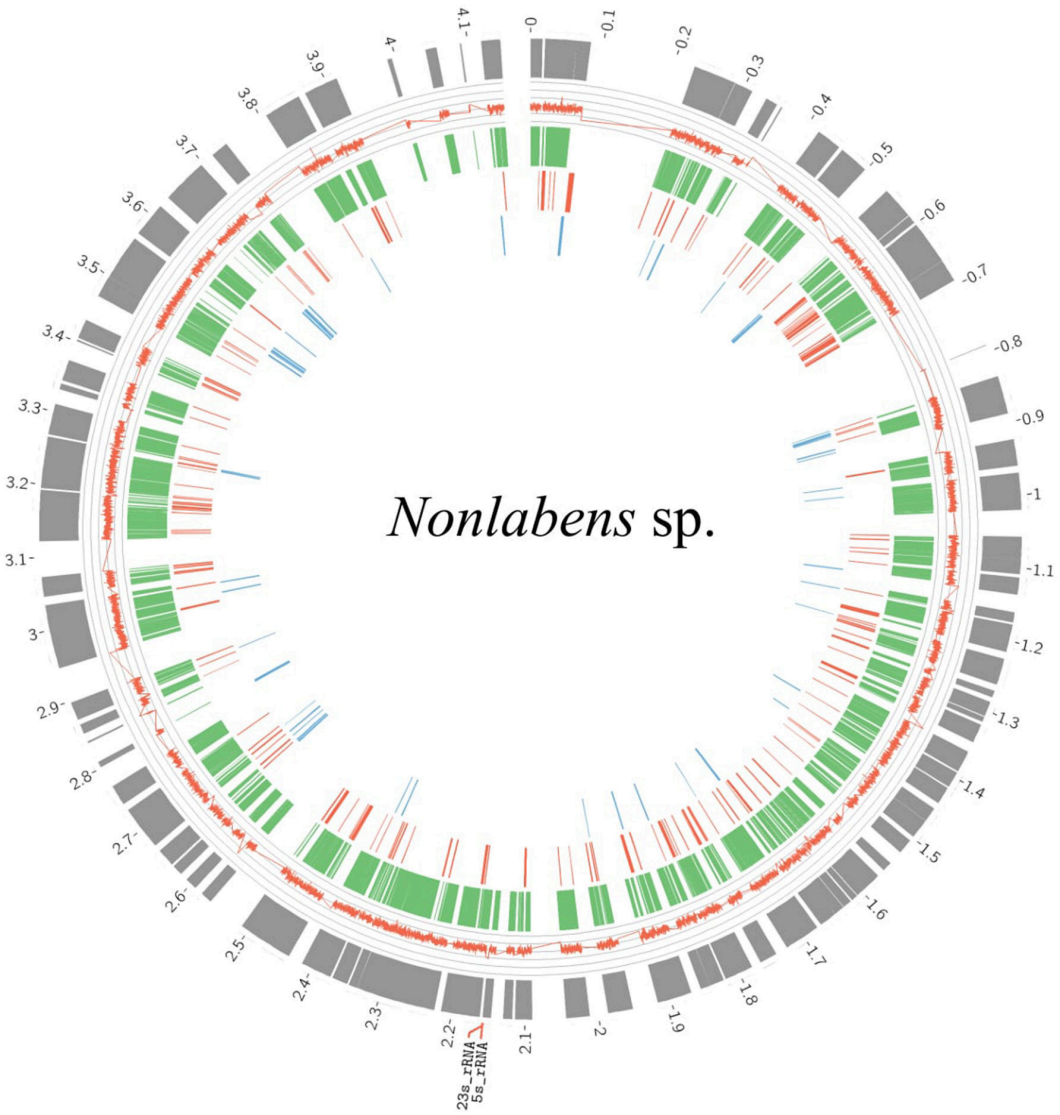
Draft genome organization of *Nonlabens* sp. *sh3vir*. and gene conservation among phylogenetically related flavobacterial reference genomes. The outermost concentric circle represents the assembled genome of *Nonlabens* sp. *sh3vir*. The green concentric circle depicts *Nonlabens dokdonensis* DSW-6, with the red and blue ones denoting the Flavobacteria bacterium BBFL7 and *Psychroflexus torquis* ATCC 700755, respectively. The completeness of draft genome of *Nonlabens* sp. *sh3vir* is about 99.8% and the size is ~ 4.2 Mb, comprising 113 contigs. The 5S and 23S rRNA are located between 2.1 and 2.2 Kb.

Flavobacteria in the viral samples at 3.0 m were verified to be distinct from those detected in the metagenomes at 3.0 m, according to two results: (1) Reciprocal BLAST analysis indicated that the flavobacteria from the viral samples (160,318 ORFs) and those from the microbial metagenomes (78,840 ORFs) shared only 2043 ORFs (0.01 and 0.03%, respectively); and (2) 16S rRNA genes predicted from both flavobacteria shared only 94% identity. Over 90% of the genome completeness of the flavobacteria in the viral samples was estimated using bacterial single-copy genes (Table S3) and up to 99.8% completeness using CheckM. Fragment recruitment analysis demonstrated that the flavobacteria had high similarity and coverage toward the genome of *Nonlabens dokdonensis* DSW-6 (Figure S4), suggesting that the identified flavobacteria in the viral samples were closely related to *Nonlabens*. The draft genome size of *Nonlabens* sp. *sh3vir*. was ~4.2 Mb, comprising 113 contigs (Figure 8), with complete gene suites for assimilatory sulfate reduction and thiosulfate oxidation as well as genes for denitrification (e.g., *nirK* and *nosZ*), nitrite reduction (e.g., *nirBD*), organic carbon oxidation, and carbon fixation.

## Discussion

We applied both 16S rRNA amplicon pyrosequencing and approaches to comprehensively explore microbial communities (bacteria, archaea, and viruses) and their potential metabolic capacity in Lake Shunet. Approximately 132 Gb of microbial and 133 Gb of viral metagenomic data were generated, which provided a much higher resolution of the microbial community structure than that in similar studies conducted in meromictic lakes (Lauro et al., 2011; Yau et al., 2013). These sequence data facilitated clustering the sequences into individual microbial bins, reconstructing a draft genome of *Nonlabens* sp. *sh3vir*. discovered in the viral samples, and generating near-complete viral genomes.

### Archaeal and bacterial diversity was high in lake shunet

Despite deep sequencing, rarefaction curves of the sequence reads plotted against OTU numbers did not reach a clear asymptote; this thus suggests that additional sequencing efforts would detect even greater diversity in this lake. Nevertheless, the observed OTU richness and diversity estimates are generally higher than those observed in other aquatic ecosystems, including two hypersaline meromictic lakes, namely the Ursu Lake (archaea *H*′ = 3.34, bacteria *H*′ = 5.45) and Fara Fund Lake (archaea *H*′ = 4.06, bacteria *H*′ = 3.74), Yellowstone Lake (Chao1 = 1074) (Clingenpeel et al., 2011; Kan et al., 2011), freshwater reservoirs (bacteria *H*′ = 5.34) (Tseng et al., 2013), and marine and mesohaline sites (archaea *H*′ = 4.2) (Hugoni et al., 2015); this is probably due to the use of different primers or sequencing depths.

### Variations of archaeal and bacterial communities were significantly explained by physicochemical parameters

Comparisons in archaeal and bacterial community compositions among Lake Shunet, Sakinaw Lake, Ursu Lake, and Fara Fund Lake suggested a unique microbial loop in Lake Shunet. However, we cannot exclude a potential error made from hypervariable regions of the 16S rRNA and the PCR bias caused by primer efficiency among studies. It might be because the environment surrounding Lake Shunet is different from the other three lakes. The observed archaeal and bacterial assemblages were dissimilar along the depths, and the variations were significantly explained by indigenous environmental parameters; these findings are in accordance with those of a study on Lake Kivu (Inceoglu et al., 2015). The sharp salinity gradient between mixolimnion and monimolimnion resulted in a lack of homogeneity of limnological parameters through stratification and likely resulted in various niches with distinct features that constituted some special microbial assemblages and metabolic capabilities (Lindstrom, 2001; Degermendzhy et al., 2010; Wear et al., 2013).

Many bacterial genera, including *Cyanobacteria, Pseudoalteromonas, Alcanivorax*, and *Rhodobacteraceae*:NA, were increased by O_2_, temperature, pH, and ${\text{NH}}_{4}^{+}$ in the mixolimnion; in this layer, key genes involved in oxygenic photosynthesis, nitrogen fixation, and nitrite reduction were detected in the bin sequences of these bacteria. At 5.5 m, H_2_S not only increased *Desulfonatronum, Desulfobacteraceae, Desulfobacterium*, and *Halothiobacillus*, which are either sulfate-reducing or sulfur-oxidizing bacteria (Castro et al., 2000; Shi et al., 2011), but also enriched Candidatus *Cloacamonas*, which is probably a syntrophic bacterium involved in anaerobic digestion (Pelletier et al., 2008) and producing H_2_S during the breakdown of organic matter.

In the archaeal community composition, *Nitrososphaera* and *Methanospirillum* were significantly explained by ${\text{NO}}_{3}^{-}$. *Nitrososphaera* is a typical ammonia-oxidizing archaeon that mediates nitrification (Spang et al., 2012), whereas *Methanospirillum* is a common syntrophic benzoate-degrading partner in benzoate degradation (Dolfing and Tiedje, 1988) and the genes involved in this degradation. The occurrence of benzoate degradation and the availability of nitrate as an electron acceptor possibly facilitated benzoate mineralization (Berry et al., 1987). This potential syntrophic relationship may have enriched both archaea at 3.0 m.

### Genes involved in energy metabolism associated with archaeal and bacterial assemblages

The potential KO profiles of energy metabolism, as well as archaeal and bacterial community compositions, differed significantly among the three sampling depths, because microbial functional profiles are usually associated with the community (Gilbert et al., 2010) or specific microbial groups (Debroas et al., 2009). For example, in the NMDS ordination, the relative abundance of *Thiocapsa* (a bacterial genus) and *Methanococcoides* (an archaeal genus) significantly explained the relative importance of potential KO involved in metabolism; specifically, *Thiocapsa* enriched K00395 (adenylylsulfate reductase, subunit B) involved in sulfur metabolism, and *Methanococcoides* enriched genes, including K00266 (glutamate synthase (NADPH/NADH) small chain), involved in nitrogen metabolism. *Thiocapsa* sp. dominated the bacterial community structure in Lake Shunet; they used hydrogen sulfide, thiosulfate, and elemental sulfur as electron donors in the anoxic chemocline zone (Caumette et al., 2004; Rogozin et al., 2012; Baatar et al., 2016). Reconstruction of elemental nutrient cycles in Lake Shunet also indicated an important role of *Thiocapsa* in sulfate reduction, consistent with a previous observation (Caumette et al., 2004). Allen et al. (2009) discovered that *Methanococcoides burtonii* isolated from Ace Lake could assimilate ammonia using a two-step glutamine synthetase (Mbur_1975) and glutamate synthase (Mbur_0092) pathway.

### Unclassified archaea and bacteria have an important role in nutrient cycles in lake shunet

Abundant untapped archaea and bacteria were detected at 5.0 and 5.5 m, respectively. This detection is similar to that in Sakinaw Lake, which had prevailing unassigned archaea in the chemocline. According to the elemental nutrient cycles of the lake that were reconstructed on the basis of binning results, several unknown or uncultured bacteria in high relative abundance (based on coverage of read counts) contributed to the elemental nutrient cycles at both 3.0 and 5.5 m. Furthermore, underexplored microbial groups were shown in various adaptive needs including bin 5.5-2, which harbored genes (*betP*/*proP*/*opuA*) involved in osmoadaptive mechanisms. The unknown archaea and bacteria were pre-dominantly diverse and played roles in nutrient cycling in this lake ecosystem.

To explore the potential roles of unclassified bacterial and archaeal OTUs (Table S4), we applied correspondence analysis to visualize the relationships between the 10 most abundant bacterial and archaeal OTUs and genes involved in energy metabolism. For example, gene K14083 (trimethylamine—corrinoid protein Co-methyltransferase, *mttB*) involved in methane metabolism was correlated with the abundance of two unknown archaeal OTUs (arcOTU_15 and arcOTU_72) and unknown bacterial OTUs (including bacOTU_14 and bacOTU_17; Figure S5). Notably, K00266 (glutamate synthase small chain) and K00262 (glutamate dehydrogenase) were potentially enriched by the specific unclassified archaea (arcOTU_31). This unknown archaeon might also assimilate ammonia using pathways similar to those in *M. burtonii* (Allen et al., 2009), suggesting that the unknown archaeon (different from all known archaeal phyla) might provide an ecological function for nitrogen assimilation in this ecosystem.

### Reconstruction of nutrient cycles in lake shunet

The observed nutrient cycles differed along the three sampling depths. For example, aerobic carbon fixation was detected only in the oxic mixolimnion, and no genes involved in sulfite oxidation were found in the chemocline, whereas nitrite oxidation was detected only in the chemocline layer. These differences were attributed to variations in the microbial community composition in the three layers, because individual microbial groups evolved to address disparate core redox reactions (Falkowski et al., 2008).

On the basis of 16S rRNA genes and ORF read counts, *Chroococcales* was the most abundant bacterial group in the oxic mixolimnion and the only biotic source of O_2_ production. Similar to Ace Lake, both the mixolimnion and monimolimnion of Lake Shunet were involved in CO production; this was due to the incomplete oxidation of organic compounds, indicating that CO oxidation might be an important pathway through which *Rhodobacteraceae* and unknown aerobic/anaerobic groups can generate energy, because numerous microbes can utilize CO for growth (Mörsdorf et al., 1992; Oelgeschläger and Rother, 2008). Because both the upper and lower layers of the lake harbored diverse and abundant bacteria and archaea in the microbial loop and might compete for organic carbon, some bacteria might obtain energy from CO. The Wood–Ljungdahl pathway of acetyl-CoA biosynthesis in this lake is in the oxic zone, which conflicts with the findings of previous studies that have suggested that this pathway is commonly found in anaerobes (methanogens and acetogens). We suggest that suspended particles in the oxic layer could create macroscale oxic–anoxic interfaces for anaerobic metabolism.

Although most of the light is absorbed by dense purple sulfur bacteria in the chemocline, we detected genes involved in light-dependent metabolism, including those in monimolimnetic assemblage. We suppose that dominant photoautotrophic *Thiocapsa* and *Cyanobacteria* were detected in the monimolimnion because they attached to the sinking particle and some weak light penetrated into the bottom layer. This echoes the results of a previous study that measured bacteriochlorophyll a in the monimolimnion of Lake Shunet (Rogozin et al., 2009).

Lake Shunet was reported to have methane concentrations of up to 32 μM (Kallistova et al., 2006); however, no genes involved in methane oxidation or methanogenesis were annotated in the current study, in contrast to previous research that has suggested that methane production commonly occurs in lake systems (Bastviken et al., 2004; Pasche et al., 2011). Our results also contrast with those reported for Ace Lake, where methane concentrations were up to 22.4 μM and genes for methane production were detected (Lauro et al., 2011). We also observed prevalent methanogenic archaea in Lake Shunet; therefore, we speculate that methane was not an important carbon source in the anoxic zone or that conditions at the time of sampling were not favorable for methane production. Moreover, the lack of methane oxidation genes could be due to zero consumption from methanotrophic bacteria, which were not identified in the lake.

Many bins in high relative abundance potentially showed the capability of nitrate reduction and nitrogen fixation, leading to the production of ammonium, which is consistent with high ammonium concentrations detected in the mixolimnion in Lake Shunet. The N_2_ source for nitrogen fixation was from the oxic zone. Complete processes of denitrification and nitrogen fixation co-occurred throughout the depths, supporting that nitrogen fixation might mainly provide bioavailable nitrogen to compensate for the loss of nitrogen by denitrification (Halm et al., 2009). In a previous study, abundant denitrifying bacteria were observed at equal levels (3.2–5.3 × 10^5^ copy mL^−1^) from the surface to the bottom of the water in the Arabian Sea; however, denitrification activity, as probed using a stable isotope tracer method, was detectable only in the middle surface—the oxygen minimum zone (Ward et al., 2009). Therefore, we inferred that the presence of denitrifying bacteria did not always activate the genes involved in denitrification. Additionally, particles such as suspended sediment in the oxic layer could create macroscale oxic–anoxic interfaces for denitrification (Jia et al., 2016).

Regarding sulfur cycling, *Thiocapsa* (bins 5-6 and 5.5-18) were the dominant purple sulfur bacteria in the lake that also utilized thiosulfate (S_2_${\text{O}}_{3}^{2-}$) instead of H_2_S as the electron donor and CO_2_ as the carbon source under anoxic conditions (Caumette et al., 2004). Annotation of genome bins revealed that not only *Thiocapsa* but also other bacteria, including *Hyphomonas neptunium*-like bacteria (bin 3-11), *Enterobacteriaceae* (bin 5-2), and *Desulfobacteraceae* (bin 5.5-12), throughout the lake harbored genes involved in reducing sulfate to H_2_S as a terminal product (Figure 4). According to the relative abundance of bins, H_2_S seemed to be produced mainly in the chemocline. The H_2_S concentration was nearly zero in both the mixolimnion and chemocline but increased sharply to 400 mg L^−1^ in the monimolimnion. Some H_2_S possibly leached from the sediment and was generated by *Thiocapsa* and *Desulfobacteraceae*; in addition, because of gravity and increasing salinity, H_2_S accumulated at the bottom of the lake from the upper layers.

Three potential chemolithoautotrophic groups, namely *Hyphomonas neptunium*-like bacteria (bin 3-11), *Enterobacteriaceae* (bin 5-5), and *Halomonas* (bin 5.5-17), were identified and found to be potentially capable of deriving carbon from carbon fixation and energy from nitrate and sulfate reduction for growth. Specifically, only bin 5-5 harbored genes for the second step of nitrification (not detected in Ace Lake metagenomes). The three potential chemolithoautotrophs all belonged to nonphototrophic proteobacteria, and this finding is consistent with the discovery of chemolithoautotrophy among ubiquitous bacterial lineages (Swan et al., 2011).

### *Nonlabens* sp. *Sh3vir*.: tiny flavobacteria discovered in a viral sample

Notably, we detected numerous flavobacterial contigs at 3.0 m when analyzing viral metagenomic data. Flavobacteria are ubiquitous, strictly aerobic bacteria, comprising diverse members (Alonso et al., 2007). According to a comparison of 16S rRNA gene sequences, these flavobacteria were different from the other flavobacteria at 3.0 m, with low similarity (only ~88%), and they had the highest phylogenetic similarity to *Nonlabens dokdonensis* (97% identity), of which cells were previously identified as *Donghaeana dokdonensis*, and the second highest similarity to *Psychroflexus torquis* ATCC 700755 (92% identity) (Yoon et al., 2006; Yi and Chun, 2012).

The reason for the detection of *Nonlabens* sp. *sh3vir*. in the viral samples warrants exploration. The cell morphology is generally rod-shaped or filamentous with a size range of 1.2–50 μm × 0.2–1.5 μm (Thomas-Jinu and Goodwin, 2004; Yoon et al., 2006; Yoshizawa et al., 2012); *Psychroflexus* are 0.2 μm in width (Bowman et al., 1998). However, the flavobacteria detected in the viral samples (< 0.22 μm) in this study were *Nonlabens* sp. *sh3vir*., which has apparently not been described. Although all known flavobacteria are larger than 0.22 μm, we speculated that this *Nonlabens* sp. *sh3vir*. was likely smaller than 0.2 μm in wide and therefore could have passed through the 0.22-μm filter. *Nonlabens dokdonensis* is strictly aerobic, which corresponds to its exclusive detection in the oxic zone 3.0 m. In addition, some of its phenotypic characteristics, including the requirement of NaCl for growth and the capability of producing H_2_S and reducing nitrate (Yoon et al., 2006), were consistent with the environmental conditions of the surface water of Lake Shunet.

In the draft genome of the tiny flavobacteria, more than 70% of the genes were similar to the reference genome of *Nonlabens dokdonensis* DSW-6 and ~3% of the genes were different, suggesting that the flavobacteria were yet to be identified *Nonlabens* sp. *sh3vir*. with a small size (<0.2 μm wide). Compared with microbial genomic bins, this genome is nearly complete; thus, we can provide more comprehensive insights into its ecological role in this lake. Moreover, this *Nonlabens* sp. *sh3vir*. is the first to be detected in an inland lake ecosystem, according to our review of the literature. The other *Nonlabens* sp. *sh3vir*p. were found only in oceanic environments.

### Viral communities were highly diverse and discriminated along the depths

In Lake Shunet, the viral assemblage included 14 major viral families (>0.1% relative abundance), exhibiting a greater diversity (*H*′: 6.70–9.15) and genotype richness (from 2,498 to 15,877). We have detected more viral families than other aquatic ecosystems, most of which had only 3–12 viral families (López-Bueno et al., 2009; Fancello et al., 2012; Tseng et al., 2013). Viral communities including *Siphoviridae, Microviridae, Myoviridae, Podoviridae, Plasmaviridae*, and *Inoviridae* were typical bacteriophages. They had different distribution patterns along the depths. For example, *Siphoviridae* were the most abundant at 3.0 m; *Microviridae*, at 5.0 m; and *Nanoviridae*, at 5.5 m. *Siphoviridae, Myoviridae*, and *Podoviridae* were distributed ubiquitously, whereas *Microviridae* and *Inoviridae* were detected in a wide range of environments, including reclaimed water (Rosario et al., 2009), the Sargasso Sea (Angly et al., 2006), and marine sediment (Breitbart et al., 2004).

So far, *Plasmaviridae* have been reported only in Antarctic lakes (Rosario and Breitbart, 2011). Certain viral types are possibly enriched by local environmental conditions through selective pressure (Angly et al., 2006; Winter et al., 2013). With the exception of bacteriophages, terrestrial eukaryotic viruses were detected in Lake Shunet—specifically, abundant *Nanoviridae* at 5.5 m. *Nanoviridae* are DNA viral pathogens of plants (Lukert et al., 1995). Blast results indicated that such plants were mostly faba bean necrotic yellows, subterranean clover stunt, and milk vetch dwarf. Because Lake Shunet has an inflow from a stream (Degermendzhy et al., 2010), terrestrial viruses could flow into the lake during the snow melt period and subsequently accumulate at the bottom of the lake, probably due to their attachment to the sinking particles. Viral reference genomes are still few, and viral communities are only presented herein based on a minority of the annotatable data. However, through the use of a nonquantitative metavironmic method, the viral assemblages in Lake Shunet can be initially described and can serve as the reference baseline or further study of meromictic lakes in Asia.

### Intimate interactions of viral, bacterial, and archaeal assemblages

The viral richness and diversity of Lake Shunet significantly differed among the depths and were associated with bacterial and archaeal richness and diversity. Therefore, we concluded that bacterial and archaeal diversity was also influenced by local viral assemblages; this is because viruses have been deemed important vehicles, shuttling genetic materials and subsequently influencing speciation of microorganisms in aquatic ecosystems (Weinbauer and Rassoulzadegan, 2003). Additionally, our results indicate that some viral taxa were specific to certain bacterial bins, echoing the notion that viruses are strain-specific predators. Consequently, high archaeal and bacterial diversity could have resulted in the high viral diversity observed (Rohwer et al., 2009). Procrustes correlation analysis revealed a significant congruence among viral, archaeal, and bacterial communities in Lake Shunet, consistent with the interactions among archaeal, bacterial, and viral populations. Microbial bins at 5.0 and 5.5 m, but not at 3.0 m, seemed to be randomly infected by viruses from both depths, we predicted that viruses attached to particles and sank into the bottom layer.

A CRISPR is an antiviral defense system common in microbial genomes (Marraffini and Sontheimer, 2010). Infection traces viral sequences incorporated in the CRISPR arrays—were detected in the microbial metagenomes of Lake Shunet, suggesting previous interactions between viral and microbial communities (Berg Miller et al., 2012). For example, CRISPRs detected at both 5.0 and 5.5 m were randomly matched to viral sequences at both depths, whereas viral sequences at 3.0 m seemed to specifically match CRISPRs at 3.0 m only. The observed infection patterns were in accordance with the patterns characterized in interactions between bins and viruses; this finding supports the assertion that both the chemocline and monimolimnion might harbor similar viral assemblages, potentially infected diverse taxonomic groups, whereas 3.0 m depth contained viruses that were more strain-specific (Rohwer et al., 2009; Atanasova et al., 2012).

### Lake shunet is a unique meromictic lake

Lake Shunet has several characteristics and physicochemical profile patterns that resemble Ace Lake in Antarctica (Laybourn-Parry and Bell, 2014). To determine the specificity of Lake Shunet in terms of microbial communities and functions, we initially performed an NMDS analysis based on embedded COG profiles obtained from microbial metagenomes of both lakes (Figure S6). In the NMDS ordination plot, Lake Shunet and Ace Lake were discriminated, indicating the existence of dissimilar microbial communities characterizing local biogeochemical processes. In addition, among meromictic lakes, Lake Shunet harbored unique prevalent bacterial and archaeal assemblages. Taken together, Lake Shunet is a unique meromictic lake in terms of microbial communities and potential functions; thus, it can serve as a baseline reference in studies of meromictic lake ecosystems, particularly in central Asia.

## Author contributions

Y-TW: 16S rRNA gene data, viral, and microbial metagenomics data analysis and integration, manuscript writing. C-YY: viral metagenomic data analysis. P-WC: sampling in the field, lab works. C-HT: partial microbial metagenomics data analysis. H-HC: sampling in the field, lab works. IS and SH: providing binning results. BB: lab works. DR and AD: sampling in the field, providing hydrological parameters. S-LT: project investigator, manuscript amendment.

### Conflict of interest statement

The authors declare that the research was conducted in the absence of any commercial or financial relationships that could be construed as a potential conflict of interest. The reviewer RZ and handling Editor declared their shared affiliation.
