# Supplementary material for: Comprehensive Insights Into Composition, Metabolic Potentials, and Interactions Among Archaeal, Bacterial, and Viral Assemblages in Meromictic Lake Shunet in Siberia
Source: Front Microbiol. 2018 Aug 20;9:1763. doi: 10.3389/fmicb.2018.01763 (PMC6109700; doi:10.3389/fmicb.2018.01763)
Supplement: Supplementary file 1 [file Table_1.DOCX]

Table S1. Summary of sequencing, assembly, and gene prediction results of microbial and viral metagenomes in Lake Shunet.
