# Supplementary material for: Comprehensive Insights Into Composition, Metabolic Potentials, and Interactions Among Archaeal, Bacterial, and Viral Assemblages in Meromictic Lake Shunet in Siberia
Source: Front Microbiol. 2018 Aug 20;9:1763. doi: 10.3389/fmicb.2018.01763 (PMC6109700; doi:10.3389/fmicb.2018.01763)
Supplement: Supplementary file 2 [file Table_2.DOCX]

Table S2. Viral sequences identified in the sequences of the respective bins. 3-1: *Bacteroidetes*, 3-2: *Flavobacteria*, 3-3: *Pseudoalteromonas*, 3-4: unknown bacterium, 3-6: unknown bacterium, 3-7: *Pseudomonas stutzeri*-like bacteria, 3-8: *Gammaproteobacteria*, 3-9: *Halomonas*, 3-10: *Rhodobacteraceae*, 3-11: *Hyphomonas neptunium*-like bacteria, 3-12: *Chroococcales*, 3-13: *Alcanivorax*, 3-14: *Verrucomicrobia*/*Chthoniobacter flavus*, 5-1: *Firmicutes*/*Clostridia*, 5-2: *Staphylococcus*, 5-3: *Bacteroidetes*, 5-4: *Bacteroidetes*, 5-5: *Enterobacteriaceae*, 5-6: *Thiocapsa*, 5.5-2: Uncultured bacterium, 5.5-3: Uncultured bacterium, 5.5-4: *Halanaerobium*, 5.5-5: Uncultured bacterium, 5.5-6: Uncultured bacterium, 5.5-9: *Deltaproteobacteria*, 5.5-10: Uncultured candidate division OP1 bacterium, 5.5-12: *Desulfobacteraceae*, 5.5-13: *Bacteroidetes*/*Marinilabiaceae*, 5.5-15: *Desulfobacteraceae*, 5.5-16: *Clostridiaceae*, 5.5-17: *Halomonas*, 5.5-18: *Thiocapsa*. Numbers denote the viral metagenome of the depth. Only the top 10 based on the contig length were shown if more than 10 viral sequences were detected.
