# Supplementary material for: Comprehensive Insights Into Composition, Metabolic Potentials, and Interactions Among Archaeal, Bacterial, and Viral Assemblages in Meromictic Lake Shunet in Siberia
Source: Front Microbiol. 2018 Aug 20;9:1763. doi: 10.3389/fmicb.2018.01763 (PMC6109700; doi:10.3389/fmicb.2018.01763)
Supplement: Supplementary file 4 [file Table_4.DOCX]

Table S4.The sequence reads of unclassified (a) bacterial and (b) archaeal OTUs without singletons at three depths in Lake Shunet.
