# Supplementary material for: Comprehensive Insights Into Composition, Metabolic Potentials, and Interactions Among Archaeal, Bacterial, and Viral Assemblages in Meromictic Lake Shunet in Siberia
Source: Front Microbiol. 2018 Aug 20;9:1763. doi: 10.3389/fmicb.2018.01763 (PMC6109700; doi:10.3389/fmicb.2018.01763)
Supplement: Supplementary file 5 [file Image_1.PDF]

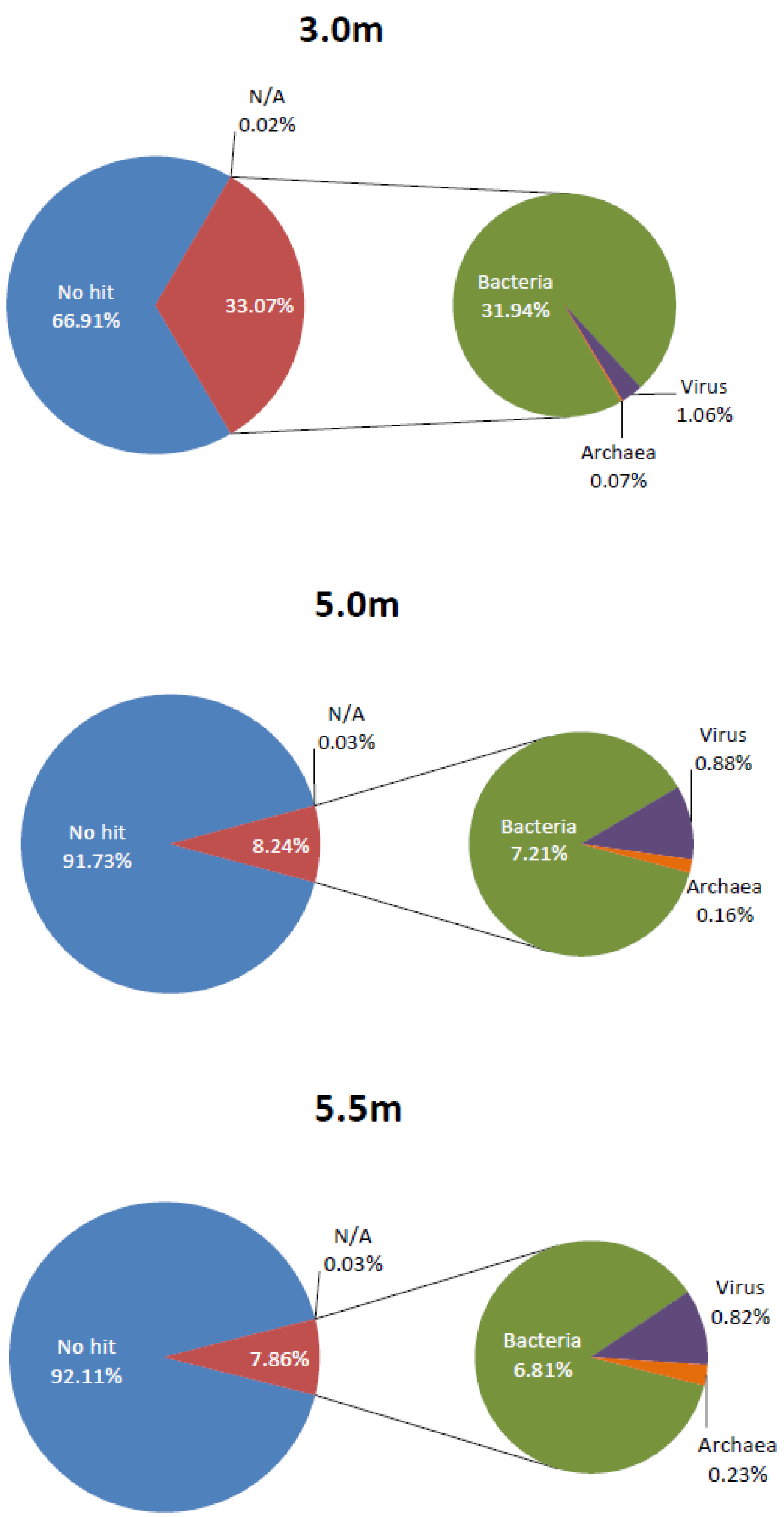

Figure S1 Distribution of BLASTp results of ORFs predicted from viral metagenomes. ORFs were searched against the NCBI RefSeq viral and microbial protein collection using BLASTp with an e-value cutoff of 10^{-5} (single best match).
