# Supplementary material for: Comprehensive Insights Into Composition, Metabolic Potentials, and Interactions Among Archaeal, Bacterial, and Viral Assemblages in Meromictic Lake Shunet in Siberia
Source: Front Microbiol. 2018 Aug 20;9:1763. doi: 10.3389/fmicb.2018.01763 (PMC6109700; doi:10.3389/fmicb.2018.01763)
Supplement: Supplementary file 6 [file Image_2.PDF]

(a)

(b)

(c)

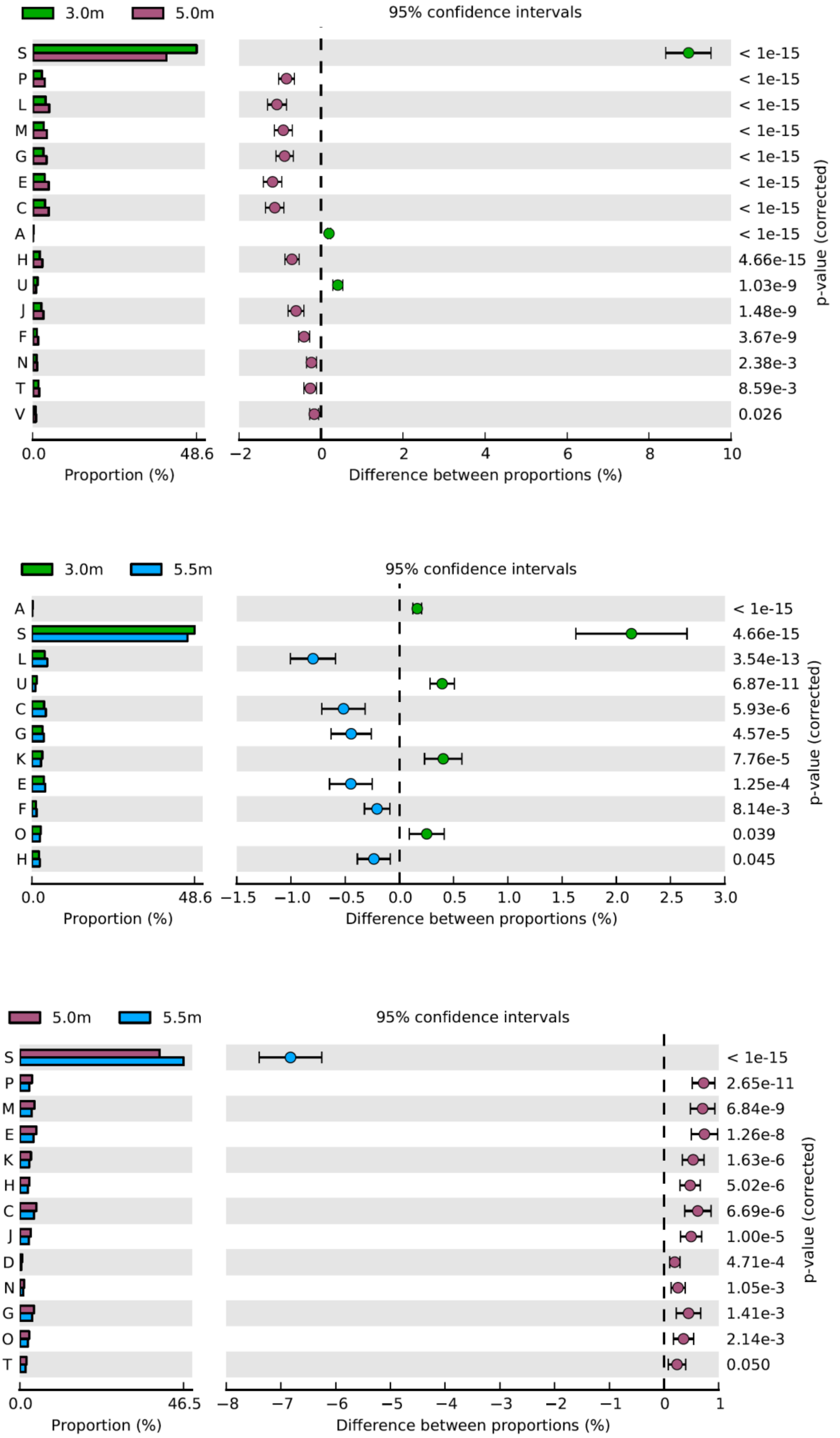
3.0m
5.0m
95% confidence intervals
S P L M G E C A H U J F N T V
< 1e-15
< 1e-15
< 1e-15
< 1e-15
< 1e-15
< 1e-15
< 1e-15
< 1e-15
4.66e-15
1.03e-9
1.48e-9
3.67e-9
2.38e-3
8.59e-3
0.026
p-value (corrected)
Proportion (%)
Difference between proportions (%)
3.0m
5.5m
A S L U C G K E F O H
< 1e-15
4.66e-15
3.54e-13
6.87e-11
5.93e-6
4.57e-5
7.76e-5
1.25e-4
8.14e-3
0.039
0.045
5.0m
5.5m
S P M E K H C J D N G O T
< 1e-15
2.65e-11
6.84e-9
1.26e-8
1.63e-6
5.02e-6
6.69e-6
1.00e-5
4.71e-4
1.05e-3
1.41e-3
2.14e-3
0.050

Figure S2 COG categories from metagenomes enriched or depleted between (a) 3.0- and 5.0 m (b) 3.0- and 5.5 m, and (c) 5.0- and 5.5 m sampling depths in Lake Shunet.. Corrected *P*-values were calculated using the Bonferroni correction. COG categories overrepresented at depth had a positive (negative) difference between proportions and are indicated by coloring. L: Replication, recombination and repair, E: Amino acid transport and metabolism, M: Cell wall/membrane/envelope biogenesis, C: Energy production and conversion, S: Function unknown, J: Translation, ribosomal structure and biogenesis, G: Carbohydrate transport and metabolism, P: Inorganic ion transport and metabolism, O: Post-translational modification, protein turnover, chaperones, T: Signal transduction mechanisms, H: Coenzyme transport and metabolism, F: Nucleotide transport and metabolism, V: Defense mechanisms, K: Transcription, U: Intracellular trafficking, secretion, and vesicular transport, D: Cell cycle control, cell division, chromosome partitioning, N: Cell motility, and A: RNA processing and modification
