# Supplementary material for: Comprehensive Insights Into Composition, Metabolic Potentials, and Interactions Among Archaeal, Bacterial, and Viral Assemblages in Meromictic Lake Shunet in Siberia
Source: Front Microbiol. 2018 Aug 20;9:1763. doi: 10.3389/fmicb.2018.01763 (PMC6109700; doi:10.3389/fmicb.2018.01763)
Supplement: Supplementary file 7 [file Image_3.PDF]

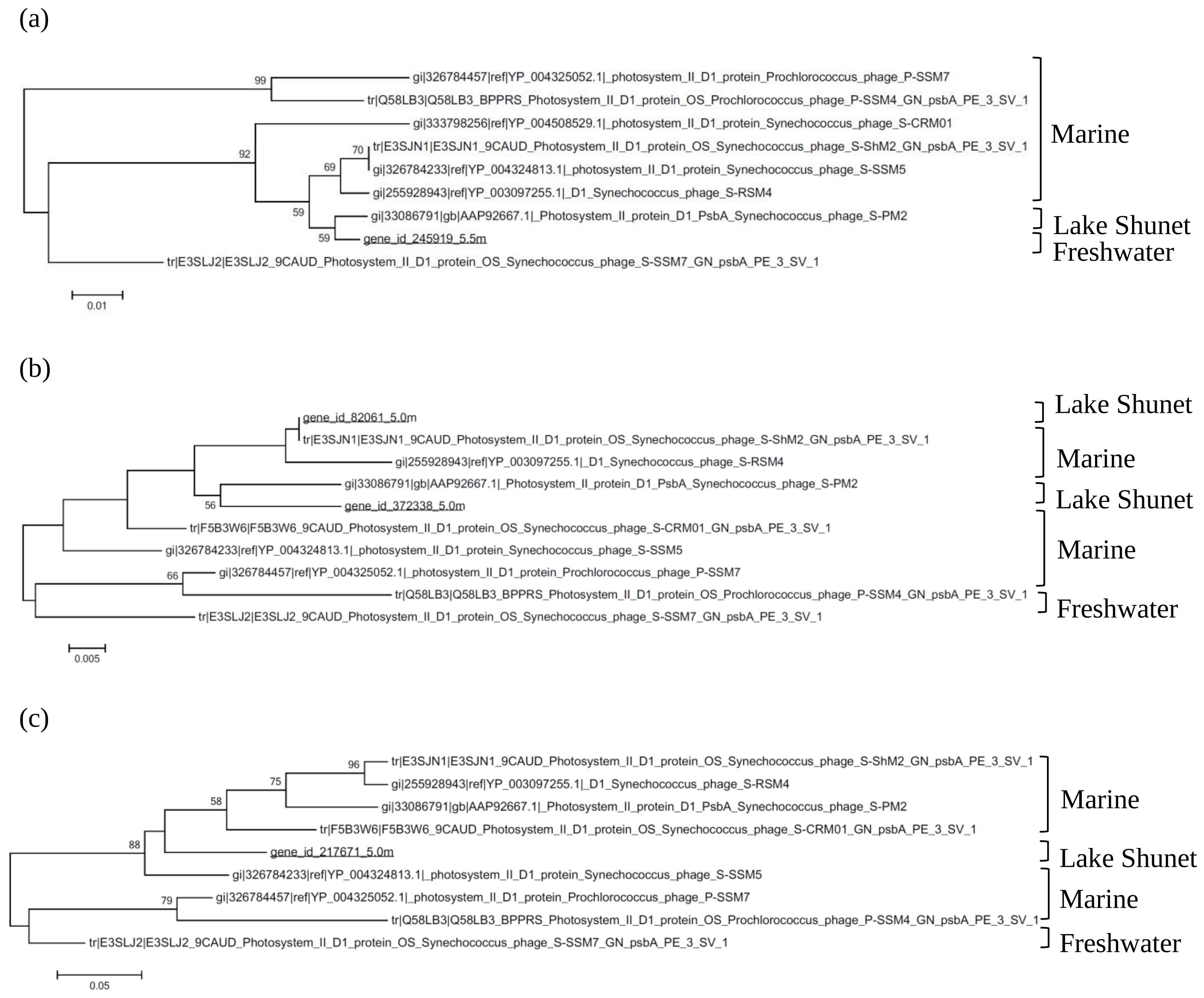

Figure S3 Phylogenetic trees of four partial *psbA* of cyanophage were constructed using neighbor-joining method separately due to no common regions among amino acid sequences (a)(b)(c). *psbA* from viral metagenomes of the study are underlined; others are obtained from UniPort and NCBI databases. Bootstrap values were calculated based on 1000 replicates and >50% are shown.
