# Supplementary material for: Comprehensive Insights Into Composition, Metabolic Potentials, and Interactions Among Archaeal, Bacterial, and Viral Assemblages in Meromictic Lake Shunet in Siberia
Source: Front Microbiol. 2018 Aug 20;9:1763. doi: 10.3389/fmicb.2018.01763 (PMC6109700; doi:10.3389/fmicb.2018.01763)
Supplement: Supplementary file 8 [file Image_4.PDF]

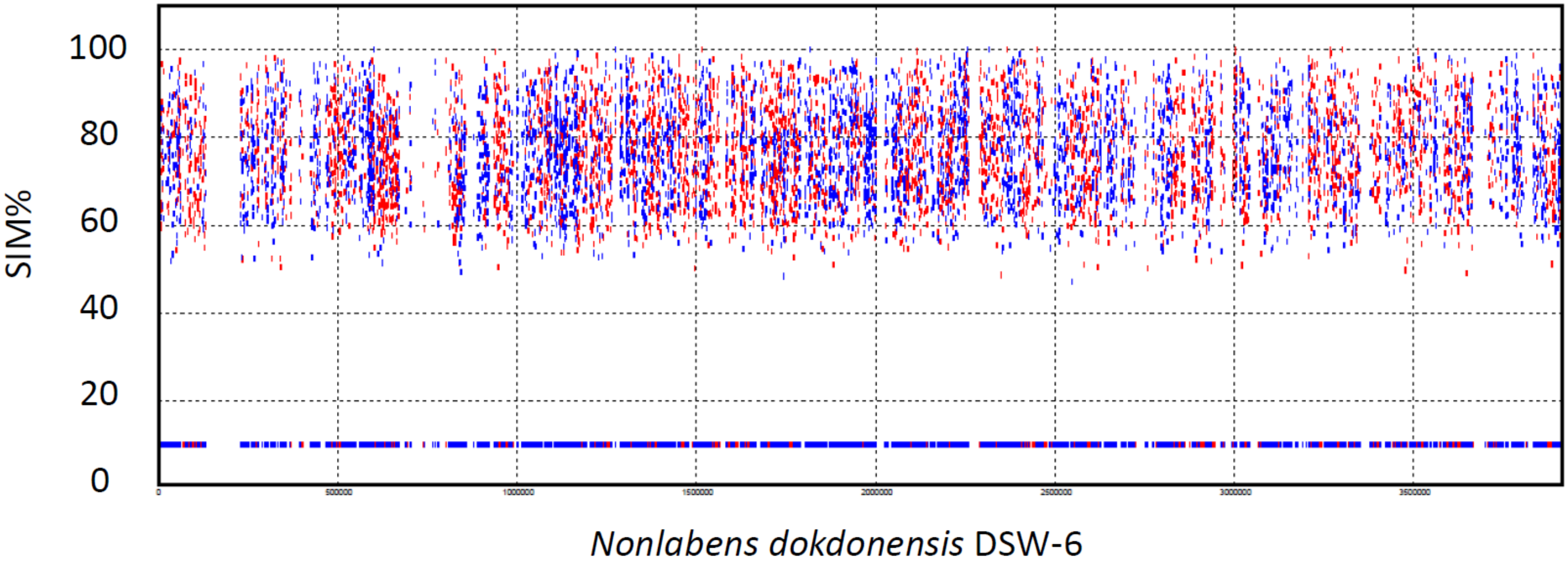

Figure S4 Fragment recruitment of the draft genome to *Nonlabens dokdonensis* DSW-6. Similarity values are based on tBLASTx alignments and coverage was plotted.
