# Supplementary material for: Comprehensive Insights Into Composition, Metabolic Potentials, and Interactions Among Archaeal, Bacterial, and Viral Assemblages in Meromictic Lake Shunet in Siberia
Source: Front Microbiol. 2018 Aug 20;9:1763. doi: 10.3389/fmicb.2018.01763 (PMC6109700; doi:10.3389/fmicb.2018.01763)
Supplement: Supplementary file 9 [file Image_5.PDF]

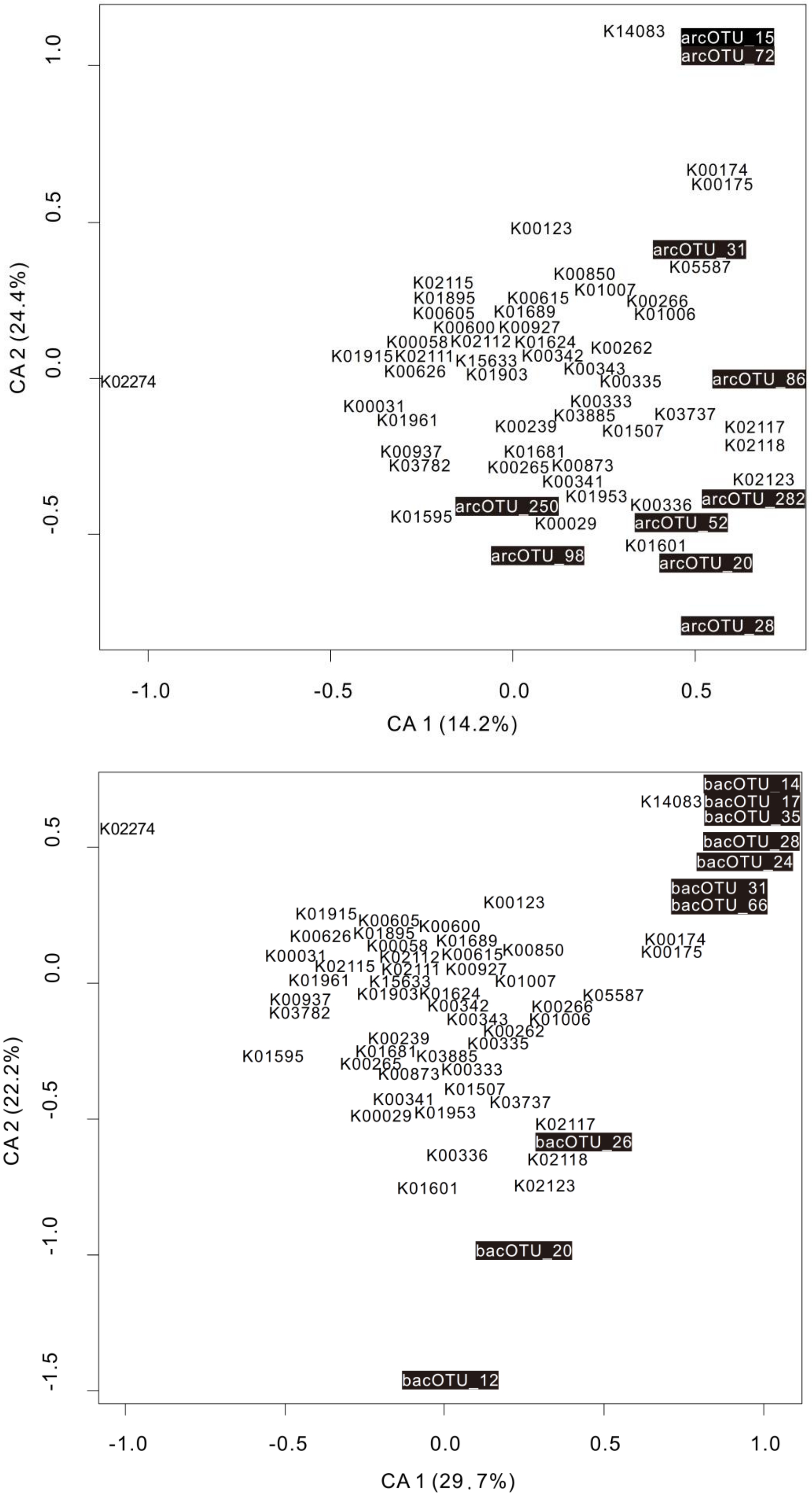

Figure S5 Correspondence analysis based on relative abundance of genes involved in energy metabolism. associated with the 10 most abundant untapped (a) archaeal and (b) bacterial

OTUs at three sampling depths in Lake Shunet. Only the 50 most abundant genes were shown on the ordination plot. **K03737** putative pyruvate-flavodoxin oxidoreductase, **K01681** aconitate hydratase 1 / homoaconitase, **K00239** succinate dehydrogenase flavoprotein subunit, **K00031** isocitrate dehydrogenase, **K00174** 2-oxoglutarate ferredoxin oxidoreductase subunit alpha, **K01903** succinyl-CoA synthetase beta subunit, **K00175** 2-oxoglutarate ferredoxin oxidoreductase subunit beta, **K00873** pyruvate kinase, **K01895** acetyl-CoA synthetase, **K01689** enolase, **K00850** 6-phosphofructokinase 1, **K00927** phosphoglycerate kinase, **K15633** 2,3-bisphosphoglycerate-independent phosphoglycerate mutase, **K01624** fructose-bisphosphate aldolase, class II, **K01915** glutamine synthetase, **K00600** glycine hydroxymethyltransferase, **K00123** formate dehydrogenase, alpha subunit, **K01601** ribulose-bisphosphate carboxylase large chain, **K00058** D-3-phosphoglycerate dehydrogenase, **K03782** catalase-peroxidase, **K14083** trimethylamine---corrinoid protein Co-methyltransferase, **K00265** glutamate synthase (NADPH/NADH) large chain, **K00266** glutamate synthase (NADPH/NADH) small chain, **K01953** asparagine synthase (glutamine-hydrolysing), **K00605** aminomethyltransferase, **K00262** glutamate dehydrogenase (NADP+), **K01507** inorganic pyrophosphatase, **K00341** NADH-quinone xidoreductase subunit L, **K02111** F-type H+-transporting ATPase subunit alpha, **K02112** F-type H+-transporting ATPase subunit beta, **K00937** polyphosphate kinase, **K03885** NADH dehydrogenase, **K00335** NADH-quinone oxidoreductase subunit F, **K02117** V-type H+-transporting ATPase subunit A, **K05587** bidirectional [NiFe] hydrogenase diaphorase subunit, **K02274** cytochrome c oxidase subunit I, **K00342** NADH-quinone oxidoreductase subunit M, **K02123** V-type H+-transporting ATPase subunit I, **K00336** NADH-quinone oxidoreductase subunit G, **K02115** F-type H+-transporting ATPase subunit gamma, **K00343** NADH-quinone oxidoreductase subunit N, **K02118** V-type H+-transporting ATPase subunit B, **K00333** NADH-quinone oxidoreductase subunit D, **K00615** transketolase, **K01006** pyruvate,orthophosphate dikinase, **K01007** pyruvate, water dikinase, **K00626** acetyl-CoA C-acetyltransferase, **K01961** acetyl-CoA carboxylase, biotin carboxylase subunit, **K00029** malate dehydrogenase (oxaloacetate-decarboxylating)(NADP+), **K01595** phosphoenolpyruvate carboxylase.
