# Supplementary material for: Comprehensive Insights Into Composition, Metabolic Potentials, and Interactions Among Archaeal, Bacterial, and Viral Assemblages in Meromictic Lake Shunet in Siberia
Source: Front Microbiol. 2018 Aug 20;9:1763. doi: 10.3389/fmicb.2018.01763 (PMC6109700; doi:10.3389/fmicb.2018.01763)
Supplement: Supplementary file 10 [file Image_6.PDF]

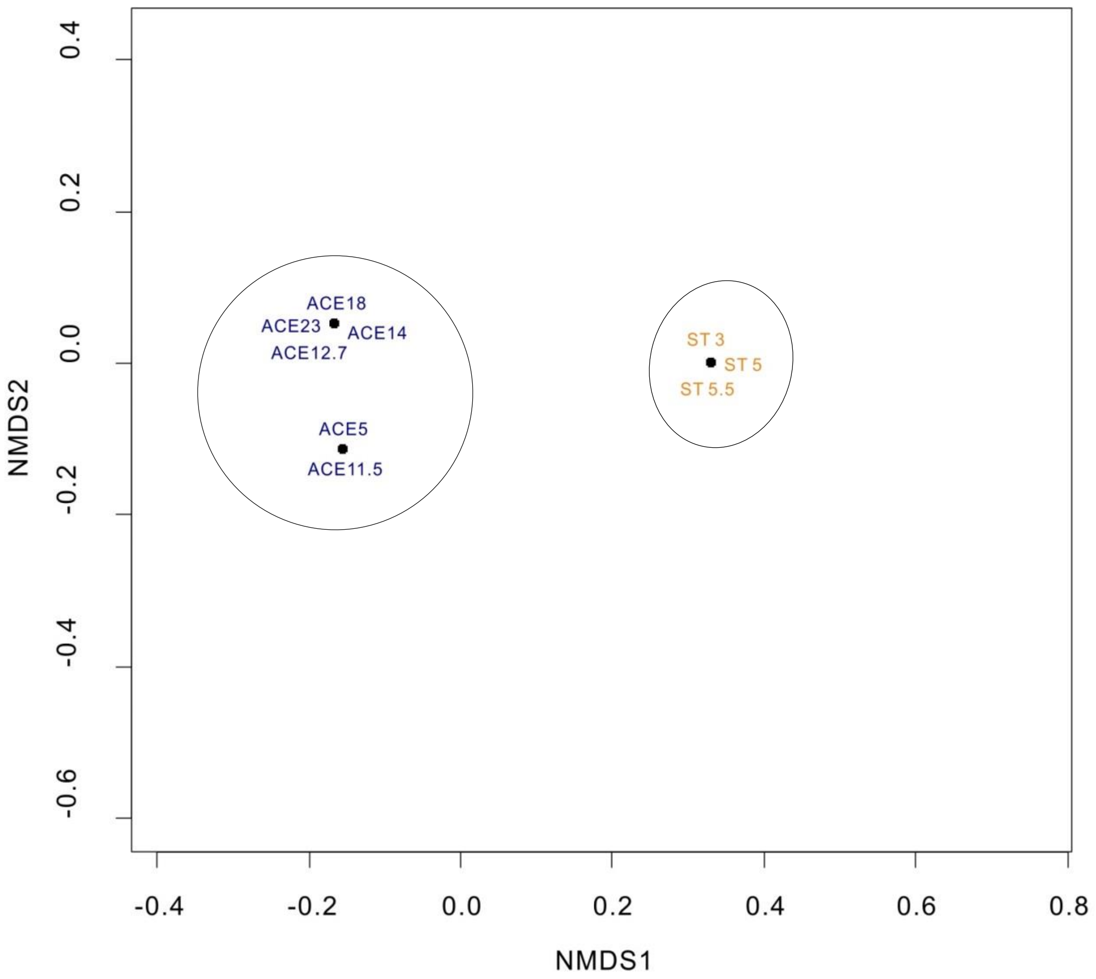

Figure S6 Nonmetric Multidimensional Scaling (NMDS) analysis based on the COG profiles obtained from Lake Shunet (0.22 µm) and ACE Lake (0.8 µm + 3.0 µm) metagenomes showed that two lakes were obviously discriminated. Numbers in labels represent the sampling depth. ST, Lake Shunet; ACE, ACE Lake.
